# Methylation-directed acetylation of histone H3 regulates developmental sensitivity to histone deacetylase inhibition

**DOI:** 10.1093/nar/gkab154

**Published:** 2021-03-15

**Authors:** Li-Yao Huang, Duen-Wei Hsu, Catherine J Pears

**Affiliations:** Department of Biochemistry, University of Oxford, South Parks Road, Oxford OX1 3QU, UK; Department of Biochemistry, University of Oxford, South Parks Road, Oxford OX1 3QU, UK; Department of Biochemistry, University of Oxford, South Parks Road, Oxford OX1 3QU, UK

## Abstract

Hydroxamate-based lysine deacetylase inhibitors (KDACis) are approved for clinical use against certain cancers. However, intrinsic and acquired resistance presents a major problem. Treatment of cells with hydroxamates such as trichostatin A (TSA) leads to rapid preferential acetylation of histone H3 already trimethylated on lysine 4 (H3K4me3), although the importance of this H3K4me3-directed acetylation in the biological consequences of KDACi treatment is not known. We address this utilizing *Dictyostelium discoideum* strains lacking H3K4me3 due to disruption of the gene encoding the Set1 methyltransferase or mutations in endogenous H3 genes. Loss of H3K4me3 confers resistance to TSA-induced developmental inhibition and delays accumulation of H3K9Ac and H3K14Ac. H3K4me3-directed H3Ac is mediated by Sgf29, a subunit of the SAGA acetyltransferase complex that interacts with H3K4me3 via a tandem tudor domain (TTD). We identify an Sgf29 orthologue in *Dictyostelium* with a TTD that specifically recognizes the H3K4me3 modification. Disruption of the gene encoding Sgf29 delays accumulation of H3K9Ac and abrogates H3K4me3-directed H3Ac. Either loss or overexpression of Sgf29 confers developmental resistance to TSA. Our results demonstrate that rapid acetylation of H3K4me3 histones regulates developmental sensitivity to TSA. Levels of H3K4me3 or Sgf29 will provide useful biomarkers for sensitivity to this class of chemotherapeutic drug.

## INTRODUCTION

Specific post-translational modifications of histone proteins are associated with gene expression. For example, methylation of histone H3 on lysine 4 (H3K4) is a hallmark of genes accessible for transcription from yeast to humans, with the lysine modified by the addition of up to three methyl groups. Within active genes, these modifications are region-specific. For example, H3K4me1 is associated with enhancer regions, while H3K4me2 and H3K4me3 are enriched at the promoter and regions proximal to the transcription start site (TSS) (1). Methylation of H3K4 is catalysed by lysine methyltransferases (KMTs) containing a characteristic SET domain (2). Another modification associated with active genes is acetylation of multiple lysine residues in the N terminal tails of histones H3 and H4. For example, H3K9 and H3K14 at the TSS region of actively transcribed genes (3) are substrates for members of the Gcn5 *N*-acetyltransferases (GNATs), MYST (Morf, Ybf2, Sas2 and Tip60) and p300/CBP acetyltransferases (4–6). These histone modifications can be removed by lysine demethylases (KDMs) and deacetylases (KDACs) to allow dynamic turnover and resetting of modifications associated with a particular gene, without histone replacement.

Aberrant levels or locations of histone marks (7) and mutations of genes encoding histone modifiers have been identified in numerous forms of cancer including mutation of KDMs and KDACs (8). A number of KDAC inhibitors (KDACis) such as suberoylanilide hydroxamic acid (SAHA) have been approved for clinical use (8) as they induce either apoptosis or differentiation of cancer cells (9). The response rate for patients is only around 30% and drug resistance can emerge during treatment (9–12). Therefore, it is crucial to gain a deeper understanding of the mode of action of hydroxamate KDACis in order to identify which cancers will respond and to characterise the mechanisms of inherent or induced resistance in order to improve the efficacy of these drugs.

Although acetylation is associated with upregulation of transcription of all genes, transcriptomic studies have found that treatment of cells with KDACis only alters the expression of a small portion of protein-coding genes (<10%) with similar numbers both up- and down-regulated (13,14). Consistent with this, histone hyperacetylation observed after KDACi treatment is not equally distributed across the whole genome (15). Early hyperacetylation preferentially occurs on histones that are already carrying the H3K4me3 modification (16). This reveals that H3K4me3 histones are normally subjected to rapid turnover of acetylation and so are the first histones to become acetylated upon KDACi treatment. This dynamic acetylation of H3K4me3 histones, targeted to H3K9 and H3K14, is conserved across evolution in human, mouse and *Drosophila* cells (17). However, it is unclear if this dynamic H3K4me3-directed acetylation is a relevant target for the long-term biological effects of hydroxamates.

Unravelling the link between H3K4me3, histone acetylation and the biological effects of KDACi is hampered by the complexity of higher eukaryotes. We have circumvented this by investigating the role of dynamic acetylation in the mode of action of the hydroxamate KDACi Trichostatin A (TSA), closely related to SAHA, using *Dictyostelium discoideum*. This is a haploid, single celled organism when feeding which undergoes multicellular development on starvation. *Dictyostelium* has many histone modifications conserved with higher eukaryotes but with a more limited number of proteins responsible for their deposition and removal (18,19). Notably, there is a single Set1 protein responsible for all detectable H3K4 methylation (20). Also, unlike mammalian genomes which contain multiple copies of genes encoding most histones, *Dictyostelium* has single copies of the genes encoding two major histone H3 variants, H3a and H3b, facilitating mutation of the endogenous genes to probe the role of histone modifications. We have shown previously that in *Dictyostelium* H3K4me3-directed H3 acetylation (H3Ac) is conserved with higher eukaryotes (21). Here, we show that loss of methylation on H3K4, either due to deletion of *Set1* or mutation of the endogenous H3 genes, changes the histone acetylation profile and confers resistance to TSA-induced developmental inhibition. We identify a *Dictyostelium* homologue of Sgf29, a reader of H3K4me2/3 found in the Gcn5 HAT complex from yeast to humans, and show that disruption of the gene leads to loss of rapid acetylation of H3K4me3 histones and also confers TSA resistance. We further demonstrate that resistance to TSA can also be achieved by overexpressing Sgf29. This highlights the dynamically acetylated pool of H3K4me3 histones as an important target involved in the biological consequences of TSA treatment and suggests that the methylation status of H3K4, as well as the levels of Sgf29, influence cellular sensitivity to hydroxamates.

## MATERIALS AND METHODS

### *Dictyostelium* cell culture, development and reagents

*Dictyostelium discoideum* (strain Ax2) cells were grown axenically in HL5 media in shaking suspension at 220 rpm at 22°C. Exponentially growing cells were used for all experiments. To assay development, 500 μl of 1.5% agar (ForMedium) in KK2 (19 mM KH_2_PO_4_, 3.6 mM K_2_HPO_4_) was added to each well of a 24-well plate and allowed to set and dry in a laminar flow hood using maximum air flow for 2h with lid open. DMSO vehicle control or TSA was applied on top of the agar to the desired concentration and the plate dried for another 2h. Exponentially growing cells were collected, washed three times with KK2 then plated on the agar, supplemented with TSA or vehicle control, at a cell density of 1.5 × 10^6^ cells/cm^2^. When applying TSA at different stages of development, cells were plated for development as above on plain agar. After 0, 2, 4, 6, 8 or 10 h of development, 20 μl of TSA (final 4 μM) or DMSO control was plated gently on top of the agar so as not to disturb the developing cells and plates allowed to dry. Addition of TSA was only possible up to 10 h when aggregates formed. In TSA pre-treatment experiments, exponentially growing cells were collected, washed and resuspend to 4 × 10^6^ cells/ml in KK2 supplemented with 4 μM TSA and shaken at 220 rpm at 22° for up to 4 h before acid extraction for histone analysis or plating for development on plain agar (1.5 × 10^6^ cells/cm^2^). During this time in non-nutrient KK2, no cell doubling is observed. Cells were allowed to develop for 24 h and pictures were taken using a camera (Hamamatsu, model ORCA-05G) attached to a dissection microscope (Leica, model MZFLLIII). The number of early and late structures were manually counted.

### Spore germination

After 48 h of development on agar in the presence of TSA as described above, fruiting bodies were harvested in KK2 containing 0.1% NP-40 and disrupted by passing through a 19G needle. The sample was enriched for spores by washing three times in KK2 (0.1% NP-40) and once in KK2. Spores were then resuspended in 1 ml KK2 and heat shocked at 45°C for 30 mins, followed by transferring 0.005% (v/v) of spores onto a *Klebsiella aerogenes* lawn on 1.5% SM agar (1% bactopeptone, 56 mM glucose, 0.1% yeast extract, 16 mM KH_2_PO_4_, 5.5 mM K_2_HPO_4_, 4 mM MgSO_4_) in a 15-cm petri dish. The number of colonies formed by germinated spores was counted manually until no new germination was observed.

### Generation of *sgf29^–^* cells

A plasmid was constructed to disrupt the *sgf29* gene by homologous recombination. The 5′ arm (1–1085 of *sgf29*) was amplified and cloned into pJET1.2 vector followed by insertion of amplified 3′ arm (1943–2877) at XhoI site. Next, the bsR cassette was obtained from the pLPBLP vector by SmaI digestion and subsequently inserted at the HincII site between the 5′arm and 3′arm, generating the final disruption vector pJET_Sgf29KO, verified by Sanger sequencing. Sequences of primers used and the map of the final construct are included in Supplementary Table S1 and Figure S8. The strategy replaces the coding sequence from 1086 to 1942 with a blasticidin resistance cassette (BsR), disrupting expression downstream of amino acid 119, whereas the TTD starts at aa 277. The disruption vector was digested with BsrGI and BstZ17I and electroporated into Ax2 cells. Following selection with 10 μg/ml blasticidin, individual resistant colonies were screened for disruption of the *sgf29* gene by PCR (Supplementary Figure S5). Three independent clones were used to verify the developmental resistance and loss of H3K4me3-directed acetylation in response to TSA.

### Cloning and purification of GST-TTD from*E*.*coli* cells

The sequence coding for the tandem Tudor domain (TTD) (S277–K411) in Sgf29 (coding for 411 amino acid residues) was amplified from cDNA. Mutation of the TTD (F359A/Y366A) was generated by two stages of PCR. Two individual fragments containing a single mutation were firstly amplified and used as templates for a second stage of amplification. Wildtype or mutant Tudor domain was then subcloned into the pGEX-KG vector using EcoRI (5′) and XhoI (3′) sites. This generated an expression cassette with GST fused to the N-terminus of the TTD and the expression in *Escherichia coli* was driven by an IPTG-inducible (Isopropyl β-d-1-thiogalactopyranoside, Sigma) promoter. All constructs were confirmed by Sanger sequencing.

Transformed *E. coli* were inoculated into 50 ml LB containing 100 mg/ml ampicillin (LB-Amp) and cultured at 37°C O.N. The starting culture was diluted into 600 ml LB-Amp the next day at 37°C until O.D 600 reached 0.55. One milliliter of the culture was stored before IPTG was added to a final concentration of 0.1 mM. The culture was then incubated at 16°C O.N. for maximum protein expression. One milliliter of the O.N. culture was stored before cells were collected and lysed by sonication (10 watts, 10s intervals with 1-minute cooling in between) in 15 ml lysis buffer (50 mM Tris, 50 mM NaCl and 1× protease inhibitor cocktail from Roche) at 4°C. Cell lysates were centrifuged at 48 000 × g for 20 min at 4°C to separate soluble protein in supernatant from inclusion bodies in the precipitates, both of which were stored for SDS-PAGE analysis. The GST-TTD fusion protein was purified using Glutathione sepharose 4B (GE Healthcare) following the manufacturer's instructions. Samples collected from various stages mentioned above, including flow through sample and purified GST-TTD fusion proteins, were resolved by 10% SDS-PAGE to follow the purification process and the purity of the final product. Sequences of primers and the final constructs used are listed in Supplementary Table S1 and Figure S8.

### Cloning and overexpression of full-length Sgf29 in *Dictyostelium*

Full length wildtype and F359A/Y366A Sgf29 were cloned by two steps. First, the coding sequence of 1–854 of *Sgf29* was amplified from cDNA. The N-terminal end of the amplicon carried a BamHI cutting site introduced by the forward primer while the C-terminal end of the fragment overlapped with the previously cloned wildtype and F359A/Y366A TTD. The N-terminal fragment was cloned into pGEX-KG carrying wildtype or F359A/Y366A TTD using BamHI and NcoI sites, generating full-length Sgf29. The full-length Sgf29 cassette also carries BglII and SpeI sites at 5′ and 3′ ends, respectively. The two sites allowed subcloning of the full-length Sgf29 cassette into the pDM304 vector containing *C*-terminal 1xFLAG tag and allows protein expression in *Dictyostelium* driven by an Actin15 promoter (22). Sequences of primers and maps of the final constructs used are listed in Supplementary Table S1 and Figure S8. All constructs were confirmed by Sanger sequencing.

### Expression of Sgf29 driven by its endogenous promoter in *Dictyostelium*

The endogenous promoter of *sgf29* (pSgf29, 824 bp upstream of start codon) was amplified and cloned into pDM304 vectors containing wildtype or F359A/Y366A Sgf29 coding sequences with a *C*-terminal 3xFLAG tag, using XhoI and BglII digestion to replace the original Actin15 promoter. The expression cassette (pSgf29-Sgf29–3xFLAG) was then subcloned into pJET_Sgf29KO to replace the 5′homology arm using the BtgI site. The BsR cassette in *sgf29*-null cells was removed by cre-lox recombination as previously described (23). The rescuing vector was digested using BsrGI and BstZ17I and electroporated into cre-loxed *sgf29* null cells. Cells surviving blasticidin selection (10 μg/ml) were pooled and evaluated for expression of 3xFLAG-tagged Sgf29 and subjected to development assays. All constructs were confirmed by Sanger sequencing.

### Acid extraction for histone enrichment

Exponentially growing cells were collected and washed three times with KK2 followed by TSA treatment for up to 4 h in shaken suspension in KK2. A total of 2 × 10^8^ exponentially growing cells were pelleted at 1700 × g for 3 min, washed in 25 ml of cold KK2 twice and lysed using 2 ml of AE lysis buffer (50 mM Tris pH 8.0, 20 mM NaCl, 3 mM MgCl_2_, 3 mM CaCl_2_, 0.5 M Sorbital 0.6% Triton X-100, 10 mM Sodium butyrate supplemented with 25 μl Phosphatase Inhibitor Cocktail 2&3 (P5726&P0044, Sigma) and 1 tablet of proteinase inhibitor cocktail (A32963, Thermo Fisher) in 5 ml lysis buffer). Nuclei were collected by centrifugation at 2500 × g at 4°C and washed twice by 1 ml of AE wash buffer (lysis buffer without salts and supplemented with 100 mM β-mercaptoethanol) at 4°C. Nuclei were then subjected to acid extraction using 250 μl 0.4M HCl for 1h at 4°C. The sample was centrifuged at 16 000 × g for 15 min to separate supernatant and insoluble proteins. The supernatant was transferred to a new 2 ml tube and the proteins were precipitated by adding 6.2× volume of cold acetone. Samples for SDS-PAGE and acid-urea (AU) gel were separated at this step with one fourth (SDS-PAGE) of the total sample transferred into one Eppendorf and the rest sample for AU gel analysis remained in the original tube. Proteins are allowed to precipitate O.N. at 4°C. Samples were washed twice with 1 ml cold acetone after O.N. precipitation. The air-dried pellet was resuspended in 1X Laemmli buffer for SDS-PAGE separation or in 8M urea, 5% acetic acid for AU gel separation.

### Protein electrophoresis and western blot of acid-extracted protein samples

Acid-Urea gel electrophoresis was carried out as previously described (17). Acid extracts were resolved by 18% SDS-PAGE or 20% Acid-urea gel then transferred to a PVDF membrane. Western analysis was carried out using antibodies against H3 (1:3000, Abcam #12079), H3K9Ac (1:3000, (17, 21)), H3bK14Ac (1:3000), H3K4me3 (1:10 000, (17, 21)), β-actin (1:3000, Santa Cruz Biotechnology #sc-47778), or FLAG (1:3000; Sigma, #F3165). Rabbit anti-H3bK14Ac antiserum was generated using the H3b-specific peptide SSQ-K(Ac)- SFPSTQGLC-KLH. Blots were imaged and band intensity quantified using an Odyssey Fc imaging system (LI-COR, Lincoln). For SDS PAGE gels the signal was normalised to that from total histone H3. The normalised signal for the 0 h sample was set as 1 and the level of modification at subsequent time points reported relative to that. The signal intensity of the H3 ladder resolved by AU gel electrophoresis was determined in ImageJ as described in Ringel *et al.* (24). Samples from at least three independent repeats were analysed.

### Statistical analysis

Results are the mean ± SD of at least three independent biological repeats unless otherwise specified. The method of statistical analysis and the correction used for individual experiments are stated in individual figure legends. Prism software was used to create graphs and perform statistical analysis.

## RESULTS

### *Dictyostelium* development is inhibited by exposure to TSA in the early stages of development

In *Dictyostelium* the developmental cycle is triggered by starvation, at which point the cells exit the proliferative cell cycle, and individual amoebae aggregate together to form a multicellular structure. This develops into a fruiting body after 24 h, consisting of a spore head raised from the surface by a stalk consisting of dead vacuolated cells. TSA inhibits *Dictyostelium* cellular KDAC activity and delays development (25). This therefore provides a quick and reliable system to assess the biological consequences of treatment with hydroxamates in a system with complexity including differentiation of different cell types, cell movement and programmed cell death.

**Figure 1.**
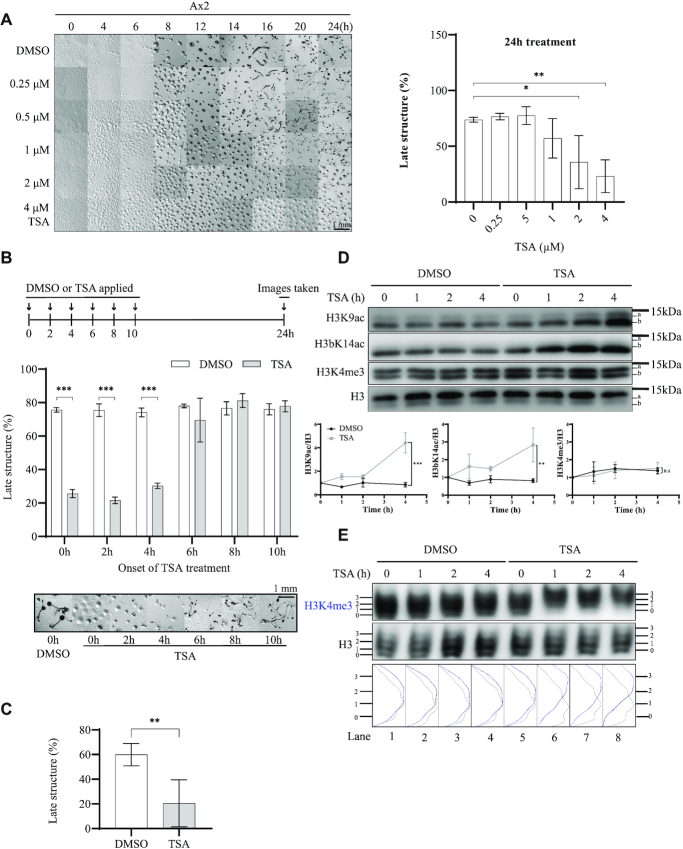
Early exposure to TSA inhibits late development of *Dictyostelium* and results in H3K4me3-directed H3 hyperacetylation. (**A**) TSA inhibits late development of *Dictyostelium*. Left panel, exponential Ax2 cells were collected, washed twice with KK2 and allowed to develop on 1.5% (w/v) agar (1.5 × 10^6^ cells/cm^2^) containing increasing concentrations of TSA (0–4 μM). Images were taken at indicated times and representative images of three repeats presented. Right panel, The percentage of late structures (beyond aggregation stage) was assessed at 24 h (average of *n* = 3 ± SD). Statistical significance was calculated using a two-way ANOVA with Tukey test. (**B**) Early exposure of cells to TSA is necessary for developmental inhibition. Upper panel, schematic illustration of the experimental design. The development assay was performed as described in A, but TSA (4 μM) was added at the indicated time during development and pictures taken after 24 h. Middle panel, quantification of late structures (average of *n* = 3 ± SD). Statistical significance was calculated using a two-way ANOVA with Tukey test. Bottom panel, representative images. (**C**) TSA pre-treatment for 4 h inhibits late development. Cells were washed with KK2 and incubated with DMSO or 4 μM TSA for 4 h in KK2 in shaking suspension at 4 × 10^6^ cells/ml and subsequently washed before plating onto agar in the absence of TSA for development for another 20 h before images were taken and the percentage of late structures calculated. Data in B and C is the average ± SD of three independent repeats and statistical significance calculated using a Student's paired T-test. (**D**) TSA-induced changes in H3K9Ac, H3bK14Ac. Cells were washed in KK2 and developed in the presence of 4 μM TSA or DMSO vehicle control for 0–4 h at 4 × 10^6^ cells/ml in KK2 before harvesting. Acid-extracts were resolved by 18% SDS-PAGE and immunoblotted using specific antibodies as indicated. Data is presented as the mean ± SD (*n* = 2). The P value was calculated using linear regression. (**E**) TSA induces H3K4me3-directed acetylation. Cells were treated and harvested as described in D. Acid-extracts were resolved on 20% acid-urea gels and immunoblotted using anti-H3K4me3 and H3 antibodies. Relative band intensity of ladders was determined using ImageJ. H3K4me3 is shown in blue, total H3 in black. One experiment representative of three is shown. *P < 0.05; **P< 0.01; ***P < 0.001.

To confirm the dose dependence and stage of inhibition, *Dictyostelium* Ax2 cells were developed by starvation on buffered agar containing increasing concentrations of TSA and analysed throughout development until vehicle-treated controls had mostly culminated to form fruiting bodies (Figure 1A). In the presence of all concentrations of TSA, aggregates formed by 8 h, a time similar to the DMSO treated controls, with aggregates actually forming more rapidly in the presence of concentrations of TSA above 0.5 μM. However a delay in culmination was apparent at concentrations of 1 μM and above. From 2 μM TSA, most of the structures observed after 24 h of development were still at the aggregate stage. Indeed, the percentage of structures past the aggregation stage (‘late structures’) after 24 h of TSA treatment decreased significantly at 2 and 4 μM compared to control cells (Figure 1A). After 48 h most structures did go on to develop fruiting bodies (data not shown) suggesting that transition from aggregation to culmination was delayed, but not permanently blocked, in the presence of TSA.

To address whether TSA is acting post-aggregation or whether the late block reflects the effects of TSA treatment earlier in development, cells were allowed to develop for 24 h on agar and DMSO or 4 μM TSA added at different timepoints (Figure 1B). All control DMSO-treated samples formed fruiting bodies at 24 h (Figure 1B). TSA-induced developmental inhibition was observed in samples where TSA was added after 0, 2 and 4 h of development but not in samples exposed to TSA after 6 h. This suggests that the block in late development is a consequence of earlier exposure to TSA. To confirm this, Ax2 cells were exposed to 4 μM TSA (or DMSO control) by developing in shaking suspension in KK2 for the first 4 h, as the cells leave the vegetative cell cycle and enter development. They were then washed to remove the TSA before being allowed to develop on agar for another 20 h in the absence of TSA. While 60% of the total structures passed the aggregate stage when cells were pre-treated with DMSO, the number dropped significantly to 20% for TSA-treated cells (Figure 1C). This confirms that treating developing cells with TSA for the first 4 h of development is sufficient to delay the appearance of late structures, suggesting that at least some of the molecular event(s) leading to developmental inhibition by TSA happen during the first 4 h.

### TSA treatment results in H3K9/K14 acetylation and preferential acetylation of H3K4me3 in developing cells

To understand the temporal change of histone acetylation during the first 4 h of development, exponentially growing Ax2 cells were starved in shaking suspension in the presence or absence of 4 μM TSA for up to 4 h. Levels of H3K9Ac, H3bK14Ac and H3K4me3 were detected by western blot, using modification-specific antisera which have been verified for specificity in *Dictyostelium* (16,17,21). The two main histone H3 variants expressed in *Dicytostelium*, H3a and H3b, can be separated on high resolution gels as H3a contains three extra amino acids (21), allowing modification of both variants to be assessed. However differences in protein sequence may alter antibody binding, hampering comparison of the levels of H3K9ac between the two variants. The antisera used to detect K14ac is specific for the H3b variant. In the absence of TSA, H3K9Ac and H3bK14Ac were apparent in developing cells and the levels did not markedly change during the first 4 h of development. However, when developed in the presence of TSA, H3K9Ac and H3bK14Ac levels increased over the time of treatment (Figure 1D). In contrast, the levels of H3K4me3 remained unchanged in control and TSA-treated cells across the 4 h. Thus, developing cells exposed to TSA in early development accumulate H3K9Ac and H3bK14Ac but no change in levels of these modifications is apparent when cells are developed in the absence of TSA.

In order to investigate the importance of preferential acetylation of H3K4me3 histones in the mode of action of TSA, it is necessary to determine if this increase in acetylation is preferentially targeted to H3K4me3 histones in developing cells as previously reported for growing *Dictyostelium* cells (21). Therefore, histone-enriched samples were resolved using acid-urea (AU) gel electrophoresis which separates proteins by charge as well as size. Methylation does not alter the charge, but acetylation of a single lysine reduces the positive charge by one, leading to a ladder of histones (level 0–3) detectable by western-blot corresponding to different acetylation states, with the least acetylated running further in the gel. The acetylation state of both total histone H3 and H3K4me3 were detected by western blot of AU gels. Consistent with the SDS-PAGE western blot analysis (Figure 1D), neither total histone H3 or H3K4me3 histones showed any increase in acetylation during the first 4 h of development (Figure 1E, lane 1–4). In contrast, cells developed in the presence of 4 μM TSA showed an upwards shift of band intensity in the H3K4me3 ladder. The major bands of the H3K4me3 ladder shifted from levels 1 and 2 to levels 2 and 3 within 1 h, while bulk histone H3 remained the same across the course of treatment revealing that the majority of histone H3 was not acetylated further (Figure 1E, lane 5–8). Taken together, TSA treatment leads to the rapid accumulation of acetylation on histone H3 in developing cells and this acetylation preferentially accumulates on H3K4me3-modified histones.

### *set1^–^* cells lacking H3K4me are resistant to TSA treatment during development

**Figure 2.**
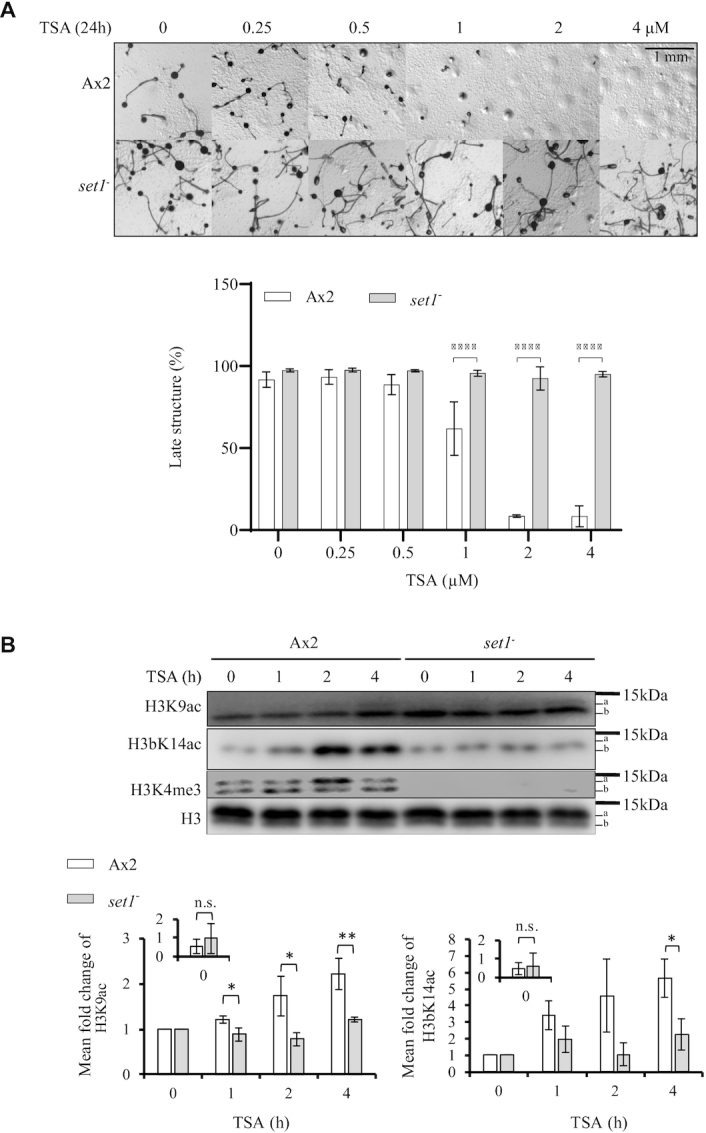
Loss of Set1 delays H3K9 and H3bK14 acetylation and confers developmental resistance to TSA. (**A**) *set1^–^* cells are resistant to TSA during development. Ax2 and *set1^–^* cells were washed with KK2 and allowed to develop on buffered agar with increasing concentrations of TSA as described in Figure 1A. Images were taken at 24 h and representative images of three repeats presented. The percentage of late structures is shown as average ± SD (*n* = 3). Statistical significance was calculated using two-way ANOVA with Tukey test. (**B**) TSA-induced increase in H3 acetylation is delayed in *set1^–^* cells. Cells were developed in the presence of 4 μM TSA for 0–4 h in KK2 before harvesting for acid-extraction as described in Figure 1. Acid-extracts were resolved by 18% SDS-PAGE and immunoblotted using specific antibodies as indicated. Levels of H3K9Ac and H3bK14Ac relative to total H3 at 0 h in each strain was determined from three biological repeats and shown as mean ± SD in the inset chart. To evaluate the increase in levels seen in the presence of TSA, the level of modification at 0h was set as 1 and other time points expressed relative to this starting point (main bar chart). Statistical significance was calculated using a Student's paired *t*-test. *P < 0.05; **P < 0.01; ****P < 0.0001.

To understand whether the rapid increase in acetylation of H3K4me3 histones is biologically relevant for TSA action, firstly the developmental sensitivity to TSA treatment of cells lacking H3K4 methylation was determined. In *Dictyostelium*, Set1 is the sole enzyme responsible for *mono*-, *di*- and *tri*-methylation of H3K4 (20). Therefore, Ax2 and *set1*^–^ cells were developed in the presence of 0–4 μM TSA (Figure 2A). As before, development of Ax2 cells was markedly inhibited, showing a significant decrease in the percentage of late structures forming at 24 h at concentrations of TSA of 1 μM or above. However *set1*^–^ cells successfully formed fruiting bodies even at the highest concentration of TSA tested, and the percentage of late structures remained similar to that of untreated cells (Figure 2A). The same resistance phenotype is apparent when treating cells with 15 μM SAHA (Supplementary Figure S1). The kinetics of histone H3 acetylation was also examined (Figure 2B). In contrast to the rapid accumulation of H3 acetylation in Ax2 cells, *set1^–^* cells failed to accumulate a significant increase in levels of H3K9Ac and H3bK14Ac upon TSA treatment during the first 4 h of development. The H3K4me3 level did not change significantly in Ax2 cells and was not detectable in *set1*^–^ cells as expected. Therefore, the effect of TSA on histone acetylation of these residues is delayed in *set1^–^* cells correlating with their resistance to TSA-induced developmental inhibition.

### H3K4A cells are resistant to TSA during development

It is known that methyltransferases containing SET domains can have substrates other than histones (26). Therefore, in order to determine whether the phenotypes of *set1^–^* cells are due to loss of H3K4 methylation, we utilized two strains in which the endogenous single copy histone H3 genes had been replaced with mutated versions to introduce an H3K4A mutation on either of the H3 variants, H3a or H3b (21). It has previously been reported that H3aK4A cells have no detectable H3K4me3 on H3a or H3b while H3bK4A cells maintain detectable H3aK4me3 but not H3bK4me3. H3aK4K cells (a gene replacement including insertion of the Blasticidin resistance cassette but without the introduced mutation) served as a control.

**Figure 3.**
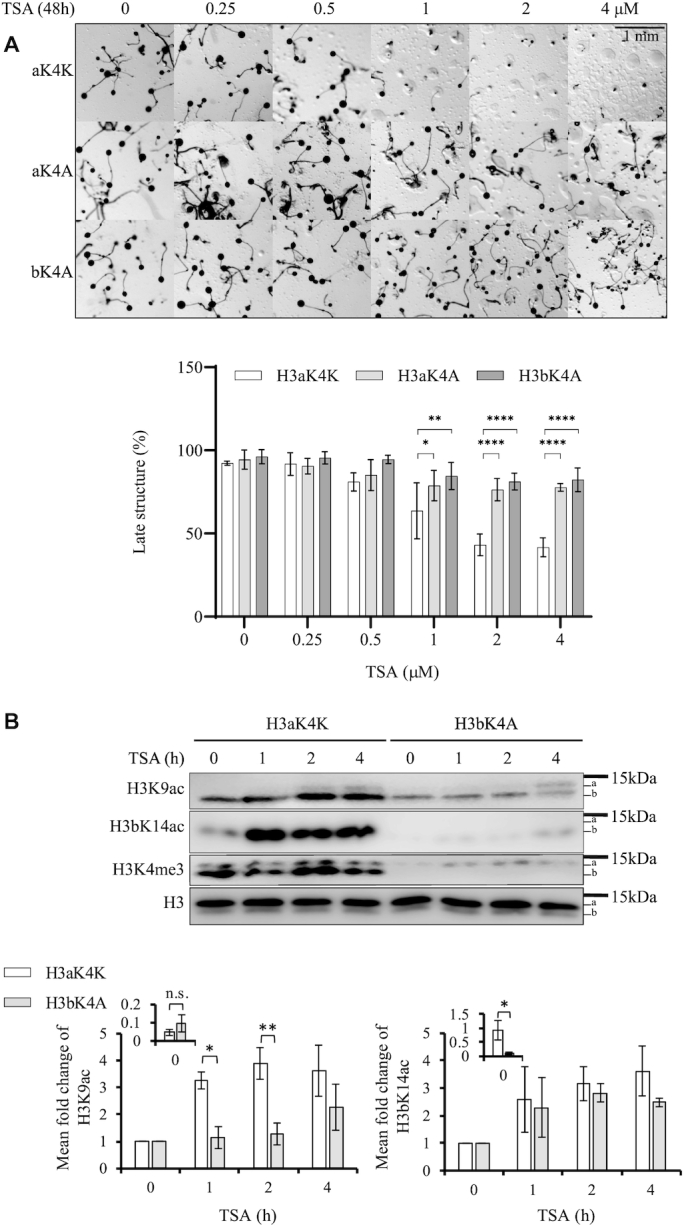
Loss of H3K4me3 delays H3 acetylation and confers developmental resistance to TSA. (**A**) H3aK4A and H3bK4A cells are resistant to TSA during development. H3aK4A, H3bK4A and control H3aK4K cells were washed with KK2 and allowed to develop on buffered agar in the presence of increasing concentrations of TSA as described in Figure 1A. Images were taken at 48 h and representative images of three repeats are presented. The percentage of late structures is shown as average ± SD (*n* = 3). Statistical significance was calculated using a two-way ANOVA with Tukey test. (**B**) TSA-induced increase of H3K9 acetylation is delayed in H3bK4A cells. Cells were washed with KK2 and developed in the presence or absence of 4 μM TSA for 0–4 h in KK2 before harvesting for acid-extraction. Acid-extracts were resolved by 18% SDS-PAGE and immunoblotted using specific antibodies as indicated. Results are presented as described in the legend to Figure 2B showing the mean ± SD (*n* = 3). Statistical significance was calculated using a Student's paired *t*-test. *P < 0.05; **P< 0.01; ****P< 0.0001.

Similar to parental Ax2 cells, development of H3aK4K cells was markedly inhibited at concentrations of TSA above 1 μM after 24 h of treatment. In contrast, H3aK4A and H3bK4A cells were able to develop beyond the aggregate stage (Supplementary Figure S2). The two K4A cells lines both required 48 h to form fruiting bodies in the presence of TSA, at which time the control H3aK4K cells still remained poorly developed with few fruiting bodies (Figure 3A). This fruiting body formation takes longer than in *set1^–^* cells that successfully formed fruiting bodies at 24 h in the presence of TSA (Figure 2A). This suggests that loss of methylation on H3K4 is at least a contributor to the resistance phenotype. Furthermore, loss of H3K4me3 on H3b alone is sufficient to confer some TSA resistance during development. Therefore, we further analyzed the change of H3 acetylation in developing H3bK4A cells exposed to 4 μM TSA (Figure 3B).

The level of H3K9Ac in H3aK4K cells was significantly increased after 1h of development in the presence of TSA (Figure 3B). Histones from H3bK4A cells showed no significant accumulation of H3K9Ac on H3b, consistent with the lack of methylation. However, on H3a (which is still detectably methylated on K4) the increase was at a similar level to that seen in control H3aK4K cells (Figure 3B). In both H3aK4K and H3bK4A cells, H3bK14Ac accumulation during development in the presence of TSA was observed. However, the overall K14Ac signal was markedly weaker in H3bK4A cells compared to H3aK4K cells, making interpretation complex. In contrast the basal levels of H3K9Ac in H3aK4K cells are similar to control cells. The level of each acetylation depends on the rate of addition and removal and, over time, the steady state levels reached which are apparently different for these two modifications. However it is not known whether the modified histones are at the same locations in the genome as in control cells. In *Dictyostelium*, the only known modification on H3K4 is methylation (19), though others cannot be ruled out. However, alongside the results from *set1*^–^ cells, this data is consistent with methylation of H3K4 being necessary for rapid accumulation of H3K9Ac following TSA treatment.

### Loss of Sgf29 delays accumulation of H3 acetylation upon TSA treatment

**Figure 4.**
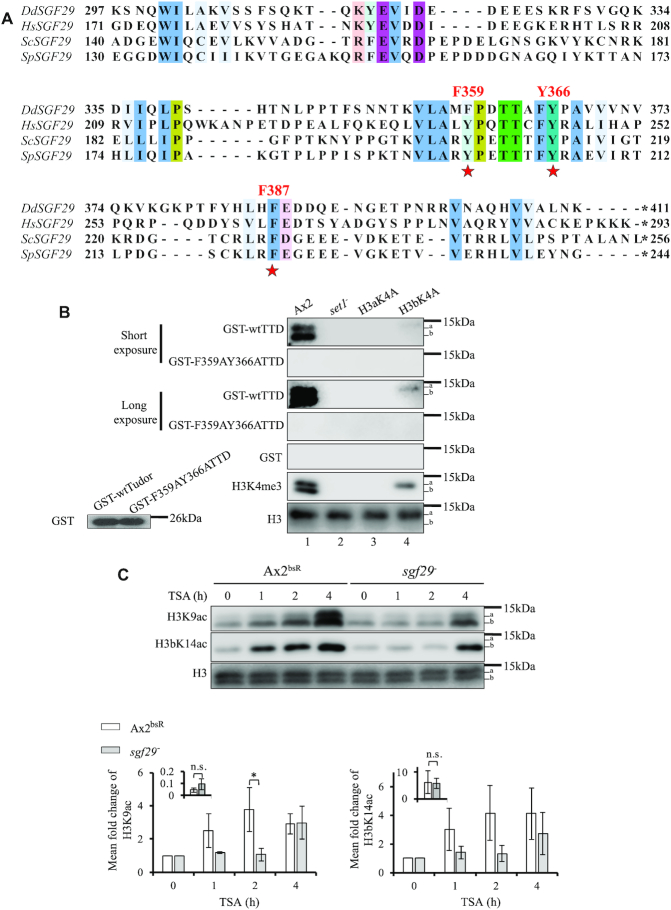
TTD of Sgf29 recognizes H3K4me3 while deletion of the gene encoding Sgf29 delays the increase in H3 acetylation on TSA treatment. (**A**) A candidate *Dictyostelium* orthologue of Sgf29 (DdSgf29, 411 a.a.) contains conserved tandem Tudor domain (TTD). Protein sequence alignment of the TTDs of Sgf29 from budding yeast (Sc), fission yeast (Sp) and human (Hs) together with DdSgf29. Conserved residues binding the tri-methylated lysine are labelled with red asterisks and their positions relative to the initiating methionine are shown. *, stop codon. Clustal X Colour Scheme is used to indicate the conservation of amino acids. (**B**) The TTD interacts with H3K4me3-modified histones. Wildtype or F359AY366ATTD was expressed and purified from *E. coli* as GST-fusion proteins. Acid extracts were prepared from exponentially growing Ax2, *set1^–^*, H3aK4A and H3bK4A cells and resolved by SDS-PAGE, transferred to a PVDF membrane and incubated with the fusion proteins as indicated. The interaction between GST-fusion protein and histone H3 is detected using an anti-GST monoclonal antibody. GST alone was used as a negative control. Equal amounts of purified recombinant proteins were used as indicated by the lower left blot. Specific antibodies were used to detect H3K4me3 and histone H3. Representative blot shown (*n* = 3). (**C**) TSA-induced increase of H3K9 and H3bK14 acetylation are delayed in *sgf29^–^* cells. Control Ax2^bsR^ cells (with random integration of the gene disruption vector) and *sgf29^–^* cells were washed with KK2 and developed in the presence or absence of 4 μM TSA for 0–4 h in KK2 before harvesting for acid-extraction. Acid-extracts were resolved in 18% SDS-PAGE and immunoblotted using specific antibodies as indicated. The mean levels ± SD of H3K9Ac and H3bK14Ac from three independent experiments are presented as described in the legend to Figure 2B. Statistical significance was calculated using a Student's paired *t*-test. *P< 0.05.

In *S. cerevisiae*, Sgf29 is a subunit of the HAT module of the histone acetyltransferase SAGA (Spt-Ada-Gcn5-acetyltransferase) complex. Sgf29 contains a tandem Tudor domain (TTD) that preferentially binds to H3K4 di*-* and tri*-*methylated histones *in vitro* and has been proposed to link H3K4me2/3 and histone acetylation (27). In order to directly test whether the H3K4me3-directed H3Ac is involved in TSA resistance by generating a strain in which H3K4me3 is conserved but its preferential acetylation is lost, we identified a candidate Sgf29 ortholog in *Dictyostelium*. The protein sequences of Sgf29 from various species share low identity in the N-terminal region but have a relatively well-conserved TTD sequence at the *C*-terminus (27). Therefore, the protein sequences of the TTD of budding yeast, fission yeast and humans were used in three independent BLAST searches against predicted *Dictyostelium* proteins (http://dictybase.org/). All three searches resulted in homology with only one candidate protein of 411 amino acids encoded by the gene DDB_G0272054, which, like Sgf29 from other organisms, is predicted to have a TTD at the C-terminus. In addition, three residues (F359, Y366 and F387) in the TTD required for interaction with *tri*-methylated lysine H3K4 in yeast (27) are conserved, though in *Dictyostelium* the first aromatic residue is a tyrosine rather than a phenylalanine (Figure 4A). A structure alignment (Supplementary Figure S3) is consistent with the TTD of the *Dictyostelium* protein being structurally conserved with that of yeast and humans (27,28).

The ability of the TTD of this *Dictyostelium* protein to interact with H3K4-trimethylated histones was tested using a far-western blot strategy. Either wildtype TTD, or a version containing two point mutations (F359A and Y366A) shown to disrupt binding to methylated H3K4 in the yeast version (27), were expressed in *E. coli* as GST fusion proteins (Supplementary Figure S4). Equal amounts of the purified protein were used to detect interaction with histone-enriched acid extracts (Figure 4B). Wildtype GST-TTD interacts with a band of the predicted molecular weight of histone H3 from Ax2 and H3bK4A cells but not *set1^–^* or H3aK4A cells which both lack the H3K4me3 modification (Figure 4B). Notably, no detectable interaction with H3b was observed in H3aK4A cells, suggesting the binding affinity of GST-TTD to H3K4me3 is stronger than H3K4me1/2, which is still detectable in these cells (21). Interaction of GST-TTD with the H3aK4me3 still present in H3bK4A cells is detectable on long exposure. In contrast, GST-F359AY366ATTD failed to detectably interact with histone H3 from any of the cells. Together, this demonstrates that the TTD interacts with H3K4me3 histones *in vitro* so this protein will be referred to as *Dictyostelium* Sgf29.

*sgf29*-null strains were created (Supplementary Figure S5) and tested for histone acetylation in response to TSA during development. Similar to previous results, control Ax2^bsR^ cells accumulated H3K9Ac and H3bK14Ac from 1 h in the presence of TSA. In contrast, accumulation of H3K9Ac and H3bK14Ac in *sgf29^–^* cells was apparent only after 4 h of development in the presence of TSA (Figure 4C). Therefore, disruption of *sgf29* in *Dictyostelium* causes delay of accumulation of H3K9 and K14 acetylation following TSA treatment during development, a phenotype shared with H3bK4A cells, but the delay is less pronounced than in *set1^–^* cells.

### *sgf29^–^* cells lack H3K4me3-directed H3Ac and are more resistant to TSA during development

**Figure 5.**
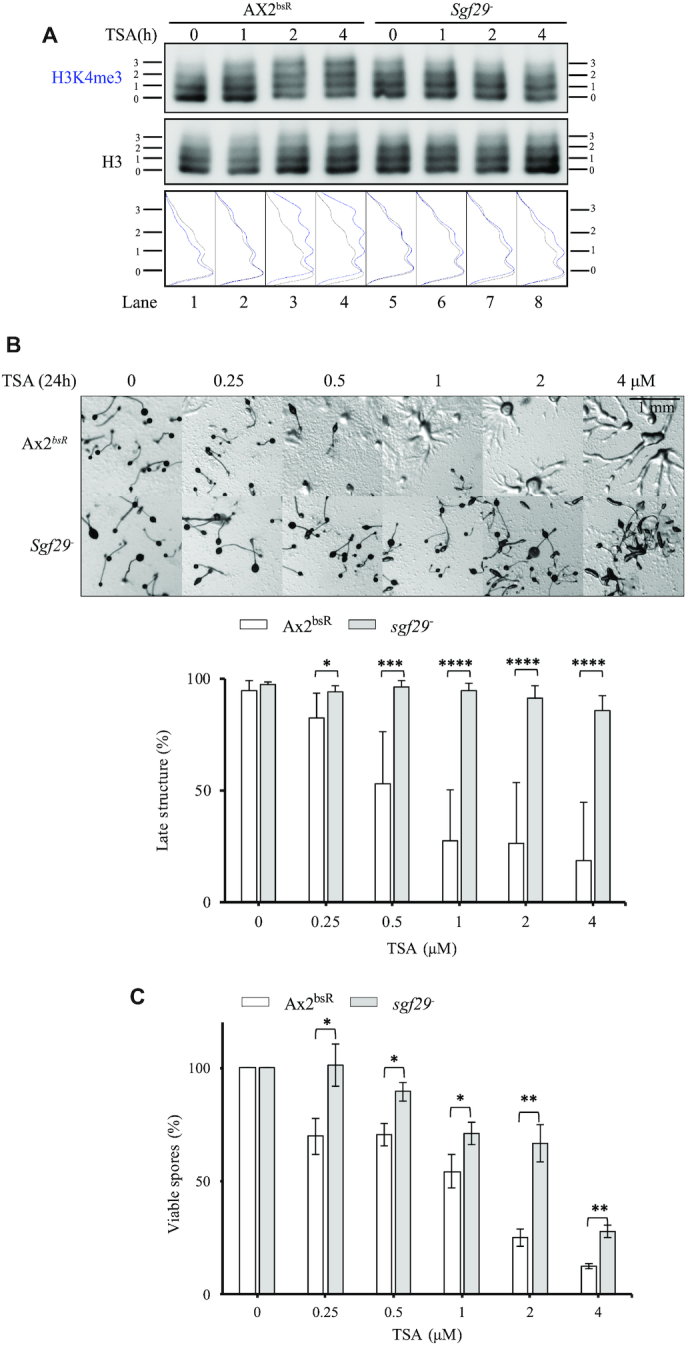
*sgf29^–^* cells have lost rapid H3K4me3-directed H3 acetylation and are resistant to TSA during development. (**A**) H3K4me3-targetted dynamic H3 acetylation is lost in *sgf29^–^* cells. Acid extracts from Ax2^bsR^ and *sgf29^–^* cells were prepared after 0, 1, 2 or 4 h of development in the presence or absence of 4 μM TSA. Samples were resolved by 20% acid-urea electrophoresis and western blots were performed using anti-H3K4me3 and anti-H3 polyclonal antibodies. The positions of histone H3 with decreasing net positive charge (0–3) are shown on both sides of each blot. One blot, representative of three independent experiments is shown. The relative band intensity of ladders was determined using ImageJ. (**B**) *sgf29^–^* cells are resistant to TSA during development. Cells were washed with KK2 and allowed to develop on buffered agar with increasing concentrations of TSA as described in Figure 1A. Images were taken at 24 h and representative images of three repeats are shown. The percentage of late structures is shown as average ± SD (*n* = 6). Statistical significance was calculated using a two-way ANOVA with Tukey test. (**C**) *sgf29*^–^ cells generate a higher number of viable spores than control cells in the presence of TSA. Spores were harvested after 48 h of development in the presence or absence of TSA and 0.005% of total harvested spores were plated on a *Klebsiella aerogenes* lawn on 1.5% SM agar in a 15-cm dish. The number of viable spores (*n* > 200 in DMSO-treated samples) was manually counted after plaque formation. Results are the average of three independent experiments: *P < 0.05; **P < 0.01, ***P < 0.001; ****P < 0.0001.

To understand if the delayed accumulation of H3 acetylation is particularly targeted to H3K4me3 histones, acetylation of histones from *Sgf29^–^* and control cells (Ax2^bsR^) were analysed by AU gel electrophoresis. As previously, more rapid acetylation of H3K4me3 than of total histone H3 was apparent in Ax2^bsR^ cells. The major H3K4me3 bands shifted from low acetylation states to high acetylation states within 2 h of TSA treatment (Figure 5A, lanes 1–4). In s*gf29^–^* cells, this rapid acetylation of H3K4me3 was delayed (Figure 5A, lane 5–8). These findings were verified in two further independently derived *sgf29^–^* clones. (Supplementary Figure S6A). Thus, Sgf29 is required for targeting preferential acetylation to H3K4me3 *in vivo* in *Dictyostelium* during development. Consistent with the importance of this acetylation, *sgf29^–^* cells are resistant to TSA during development (Figure 5B, Supplemental Figure S6B for independent clones). These cells are also resistant to SAHA-induced developmental inhibition (Supplementary Figure S6C). To quantify this further the number of viable spores after development in the presence of TSA was assessed. Control Ax2^bsR^ cells showed a decrease in the number of viable spores in the presence of increasing concentrations of TSA and *sgf29^–^* cells generated significantly higher numbers of viable spores at all concentrations tested (Figure 5C). To rule out any potential defect in proliferation being causative of the phenotypes seen in *sgf29^–^* cells, we compared the growth curves of in Ax2^bsR^, *set1^–^* and *sgf29^–^* cells grown in shaking suspension and did not see any significant differences in our culture conditions (Supplementary Figure S7).

Therefore, disruption of the gene encoding Sgf29 not only results in delay of histone acetylation and loss of rapid H3K4me3-directed H3Ac, but also makes cells resistant to hydroxamate-induced developmental inhibition (Figures 4 and 5). These results support the hypothesis that Sgf29 mediates the crosstalk between H3K4me3 and histone acetylation, and that crosstalk is important for the mode of action of TSA.

### The role of Sgf29 in TSA sensitivity is dependent on the tandem Tudor domain

**Figure 6.**
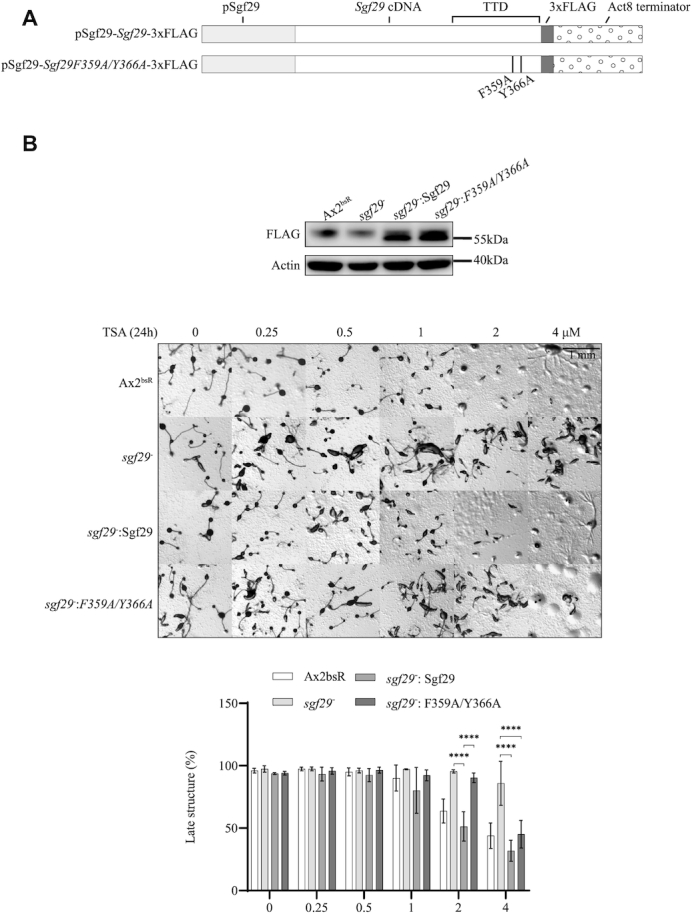
Developmental sensitivity to TSA depends on a functional TTD of Sgf29. (**A**) Schematic illustration of wildtype and F359A/Y366A Sgf29 expression constructs. The expression of *C*-terminally 3xFLAG-tagged protein is driven by the endogenous promoter of *sgf29* in an integrating vector. (**B**) Expression of Sgf29–3xFLAG in *sgf29*^–^ cells re-sensitizes cells to TSA during development. Upper panel, immunoblots showing expression of 3xFLAG-tagged wildtype and F359A/Y366A Sgf29 in *sgf29^–^* cells. Whole cell lysates control *sgf29^–^* cells and *sgf29^–^* cells expressing wildtype or F359A/Y366A Sgf29–3xFLAG was resolved by 12% SDS-PAGE and blotted using anti- FLAG antisera. Actin is used as a loading control. Lower panel, cells were washed with KK2 and allowed to develop on buffered agar with increasing concentration of TSA as described in Figure 1A. Images were taken at 24 h and representative images of three repeats shown. The percentage of late structures is shown as average ± SD (*n* = 3). Statistical significance was calculated using a two-way ANOVA with Tukey test. ****P < 0.0001.

To confirm that the phenotypes seen above were due to loss of Sgf29 expression and to determine whether this was dependent on the ability of the protein to bind H3K4me3, constructs were generated to drive expression of epitope-tagged full length Sgf29–3xFLAG or Sgf29F359A/Y366A-3xFLAG from the endogenous *sgf29* promoter (containing sequences 831 bp upstream of the start codon) (Figure 6A). Single copy insertion required blasticidin as a selectable marker so the BsR cassette used to create the gene disruption in *sgf29^–^* strain was first removed using the cre-lox system as previously described (23). Expression of similar levels of Sgf29 or Sgf29F359A/Y366A was confirmed using anti-FLAG-antibody (Figure 6B). Expression of Sgf29 in *sgf29^–^* cells re-sensitizes cells to TSA during development to a level similar to Ax2^bsR^ cells (Figure 6B). At concentrations above 2 μM TSA, the percentage of late structures of *sgf29^–^* cells was significantly higher than cells re-expressing Sgf29. In the contrast, the number of late structures remained high at 90% on 2 μM TSA treatment in cells expressing Sgf29F359A/Y366A but at 4 μM TSA the number of late structures dropped significantly to 45% compared to *sgf29^–^* cells (Figure 6B, lower panel). The result confirms that loss of Sgf29 is responsible for the TSA resistance in *sgf29^–^* cells while also suggesting that the mutated Sgf29F359A/Y366A still has a partial function *in vivo*.

### Overexpression of Sgf29 also leads to TSA resistance during development

**Figure 7.**
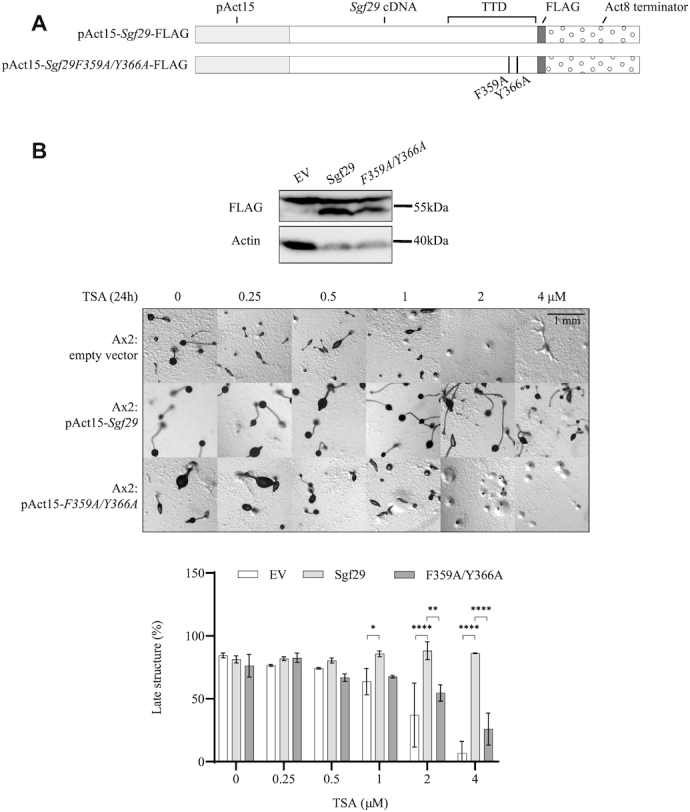
Overexpression of Sgf29-FLAG confers resistance to TSA during development. (**A**) Schematic illustration of wildtype and F359A/Y366A Sgf29-FLAG overexpression constructs. The expression of *C*-terminally FLAG-tagged protein is driven by the actin 15 promoter. (**B**) Overexpressing Sgf29-FLAG in Ax2 cells confers resistance to TSA during development. Upper panel, immunoblots showing expression of FLAG-tagged wildtype and F359A/Y366A Sgf29 in Ax2 cells. Whole cell lysates from pools of cells transfected with empty vector (EV), or constructs to drive expression of wildtype and F359A/Y366A Sgf29 were resolved by 12% SDS-PAGE and blotted using an anti-FLAG antibody. Actin is used as a loading control. Lower panel, cells were washed with KK2 and allowed to develop on solid agar with increasing concentration of TSA as described in Figure 1A. Images were taken at 24 h and representative images of three repeats shown. Lower panel, the percentage of late structures is shown as average ± SD from three independent experiments. Statistical significance was calculated using a two-way ANOVA with Tukey test. *P < 0.05; **P < 0.01; ****P < 0.0001.

As a number of tumour cells show increased levels of Sgf29 (COSMIC database COSU376, COSU377 and COSU414) we tested whether overexpression would also alter TSA sensitivity. Constructs to drive overexpression of full length Sgf29-FLAG or Sgf29F359A/Y366A-FLAG, driven by the strong actin 15 promoter using an extrachromosomal, multicopy vector (Figure 7A), were introduced into Ax2 cells and expression confirmed. (Figure 7B). The development of Ax2 cells overexpressing Sgf29 was markedly more resistant to TSA than control cells. In addition, Ax2 cells overexpressing Sgf29F359A/Y366A-FLAG exhibited mild resistance with the formation of late structures significantly inhibited down to 26% in the presence of 4 μM TSA compared to 7% in cells with empty vector and 86% in cells overexpressing Sgf29-FLAG (Figure 7B). These results suggest that the level of Sgf29 expression defines the sensitivity of response to TSA as either its loss or overexpression leads to TSA resistance.

## DISCUSSION

Despite the use of compounds with KDACi activity in treatment of diseases such as cancer, the molecular mechanisms by which these inhibitors bring about long-term biological effects is unclear. We identify the rapid, methylation-directed acetylation of K9 and K14 on histone H3 as an important pool of histones mediating the sensitivity of *Dictyostelium* development to KDACi.

KDACs have been implicated in gene expression changes associated with a number of developmental processes. For example in haematopoiesis, lineage-specific gene expression requires multiple KDACs (9). Inhibition of KDACs by SAHA pre-treatment or deletion of KDAC3 in mouse hematopoietic stem cells results in loss of self-renewal, indicative of loss of multilineage potential (29). In *Dictyostelium*, TSA treatment causes more rapid initial development up to aggregate formation, a phenotype shared *set1^–^* cells (20). However TSA delays later stages of development with reduced numbers of fruiting bodies apparent after 24 h and a reduction in the number of viable spores generated after 48 h. Exposure of cells during the first 4 h following starvation is necessary and sufficient to confer inhibition, and also leads to an increase in H3K9 and H3bK14 acetylation. Importantly, this acetylation is more rapidly increased on histone H3 molecules already containing the H3K4me3 modification. This rapid turnover of H3 acetylation on H3K4me3 histones has been reported in mouse, *Drosophila* and human cells (17) and we have previously reported that this is conserved in proliferating vegetative *Dictyostelium* cells (21). This is the first report that this rapid turnover is also found in cells entering differentiation. In human cells this dynamic acetylation of H3K4me3 histones is targeted to both H3K9ac and K14Ac (17). This, and the availability of specific antibodies that recognise these sites in *Dictyostelium* histones (21) led us to study these modifications. Changes in acetylation on other lysine residues could also be linked to H3K4me3 status, as yeast GCN5 mediates acetylation on at least six lysines in histone H3 (30). The complete shift of *Dictyostelium* H3K4me3 to higher bands on AU gels following TSA treatment (e.g. Figure 1E) would suggest that this might be the case.

The phenotypes of *set1^–^* and H3K4A strains are consistent with dynamic acetylation of H3K4me3 being relevant for the biological action of TSA (20,21). The H3aK4A gene replacement strain lacks any detectable tri-methylation of H3a or H3b, although some mono- and di-methylation of H3b is apparent (21). Conversely, H3bK4A cells do still have detectable H3aK4me3, so these strains distinguish the role of trimethylation from mono- and di-methylation, as well as importance of the two histone variants. No strain is available that only blocks methylation of H3a, so it cannot be ruled out that there is a consequence of the linked modifications on this variant. However in all strains there is correlation between H3bK4me3, rapid H3K9 acetylation and resistance to the developmental block induced by TSA, lending support to the simplest explanation that the methylation on K4 directs dynamic acetylation and this turnover has biological consequences for resistance to TSA during development.

It could be argued that changes in gene expression caused by loss of H3K4me3 are the root cause of developmental resistance to TSA, not the consequent dynamic acetylation. Therefore, we generated a strain in which the methylation is preserved but the link between H3K4me3 and H3 acetylation is broken. In *S. cerevisiae*, the Sgf29 protein provides such a link as it binds H3K4me3 and is associated with the GCN5 HAT complex. The protein coded by DDB_G0272054 is a strong candidate for the *Dictyostelium* orthologue of Sgf29 as it is the only protein encoded in the genome with a TTD. This TTD interacts *in vitro* specifically with H3K4me3 and not with histones lacking this mark, and is dependent on residues in the TTD required for this interaction in yeast (27). The binding of recombinant protein to H3b in H3aK4A cells is undetectable, even though H3K4me1/2 is still detectable on H3b in these cells (21). The *C*-terminal TTD of both human and budding yeast Sgf29 favours binding to H3 peptides containing H3K4me3 over H3K4me2 although both interactions are detectable (27). H3K4me3 has been shown to be the preferred ligand and Sgf29 is localised at gene promoters, overlapping with the H3K4me3 modification (31). Any TTD binding to H3K4me2 histones is below the detectable limit in our analysis (21).

As in other organisms, disruption of the *sgf29* gene is not lethal (27,32) and is not required for *Dictyostelium* development. In *C. neoformans*, disruption of *sgf29* leads to hypervirulence, and inability to form titan cells, and in Arabidopsis to salt tolerance (32–34). Importantly disruption of the *Dictyostelium sgf29* gene successfully abrogates rapid accumulation of H3K9/14 acetylation and loss of rapid acetylation of H3K4me3 histones upon TSA treatment. The kinetics of acetylation of H3K4me3 are not distinguishable from that of bulk H3. In future work it will be important to determine the genetic loci at which these modifications are found and investigate the association of Sgf29 with these loci to unravel the molecular basis of the developmental effect. This agrees with a previous finding that loss of the TTD in *S. pombe* Sgf29 results in loss of acetylation of H3K4me3 nucleosomes *in vitro* but the reactivity of HAT module remains the same (24). The basal level of H3K9 and K14 acetylation in *sgf29^–^* cells is similar to control cells which differs from the situation in *S. cerevisiae* where loss of Sgf29 leads to a reduction in global acetylation levels (27).

Overall our data is consistent with the identified protein being the orthologue of Sgf29 and that in *Dictyostelium* it plays a role in the sensitivity of development to TSA, as gene disruption confers resistance. Mutation of the TTD at residues F359 and Y366 was sufficient to lose *in vitro* binding activity but cells expressing full length protein with the equivalent mutations show an intermediate resistance phenotype, suggesting some residual binding activity *in vivo* or another chromatin targeting domain in the complex. This is consistent with *S.cerevisiae* Sgf29 where mutation of individual binding residues reduced H3 acetylation but not to the level of complete deletion of Sgf29 (27).

Overexpressing Sgf29 in Ax2 cells also leads to resistance to TSA. One explanation could be that free excess protein binds to H3K4me3 and blocks recruitment of the acetyltransferase complex. This raises the interesting possibility that either loss or overexpression of Sgf29 in tumour cells can act as a therapeutic biomarker for potential resistance to hydroxamates. In the COSMIC database (https://cancer.sanger.ac.uk/cosmic), over 10% of tested patient samples of breast, haematological, large intestine and stomach cancers show overexpression of Sgf29 as well as 5 out of 8 cancer cell lines, compared to non-transformed lines (35). This overexpression has been proposed to lead to upregulation of expression of the oncogene c-myc, for example in hepatocellular carcinomas (36). It will be informative to investigate whether these cells show altered sensitivity to hydroxamates and whether other components of this pathway such as reduced levels of H3K4me3 seen in some cancers, could also provide biomarkers for resistance (37,38).

## DATA AVAILABILITY

Dictybase is a database hosting whole genome sequence of *Dictyostelium* species and web tools such as BLAST (http://dictybase.org/).

Data of overexpression of Sgf29 in samples from patients with breast, haematological, large intestine and stomach cancers can be found in COSMIC database (https://cancer.sanger.ac.uk/cosmic).

Protein structure analysis and comparison can be performed using Chimera (https://www.cgl.ucsf.edu/chimera/).

## Supplementary Material

gkab154_Supplemental_File

## Notes

Present address: Duen-Wei Hsu, Department of Biotechnology, National Kaohsiung Normal University, Kaohsiung City 82444, Taiwan.
