# Supplementary material for: Methylation-directed acetylation of histone H3 regulates developmental sensitivity to histone deacetylase inhibition
Source: Nucleic Acids Res. 2021 Mar 15;49(7):3781–95. doi: 10.1093/nar/gkab154 (PMC8053100; doi:10.1093/nar/gkab154)
Supplement: gkab154_Supplemental_File [file gkab154_supplemental_file.pdf]

Fig. S1. Development of Ax2 and *set1*⁻ cells after 24 hours of 15 μM SAHA treatment.
Exponentially growing Ax2 and *set1*⁻ cells were collected and washed twice with KK2 then transferred onto 1.5% agar (1.5 × 10^6 cells/cm^2) containing 15 μM SAHA or DMSO vehicle control. Images were taken at 24 hours and representative images of two repeats are presented. Scale bar represents 1 mm.

Fig S2. Development of H3aK4A and H3bK4A cells after 24 hours of TSA treatment.
Exponentially growing H3aK4A, H3bK4A or control H3aK4K cells were collected and washed twice with KK2 then transferred onto 1.5% agar (1.5 × 10^6 cells/cm^2) containing increasing concentrations of TSA or DMSO vehicle control. Images were taken at 24 hours and representative images of three repeats are presented. Scale bar represents 1 mm.

Fig S3. Structure alignment of the TTD of *Dictyostelium* Sgf29 to budding yeast and human counterparts.
The predicted tertiary structure of the TTD of *Dictyostelium* Sgf29 is shown by a blue ribbon while that of human (HsSGF29) or budding yeast (ScSGF29) is shown in grey. Conserved binding residues in *Dictyostelium* Sgf29 are shown as purple sticks. K4-trimethylated lysine is represented as a green stick. Structural prediction and comparison were done using Chimera (https://www.cgl.ucsf.edu/chimera/).

Fig S4. Purification of GST-TTD fusion proteins following expression in *E.coli*.
GST-fusion proteins were purified using glutathione sepharose. Samples from stages of the purification were resolved by SDS-PAGE and proteins were visualized by staining with Coomassie blue for **A.** GST-TTD (42 kDa), **B.** GST-F359AY366ATTD (42 kDa) and **C.** GST (29kDa) in individual gels.

Fig S5. Generation and genotyping of *sgf29*⁻ cells
A. Schematic representation of the gene replacement event to generate *sgf29*⁻ cells. Exon, intron and intergenic regions are represented by a thick black solid line, a dashed line and a solid grey line respectively. The TTD, marked yellow, is located at the 3'end of the coding region. The 5' arm spanning 1 to 1084 relative to the start codon, 3' arm (1943 to 2877) and the BsR cassette was cloned into pJET1.2 vector to generate pJET_sgf29KO disruption vector.

Specific primer sets were designed to detect bsR insertion (P1 & P2; P3 & P4). A primer set (P1 & P4) flanking the linearized fragment was used to detect parental contamination. **B.** Map of the pJET_sgf29KO disruption vector. The vector was digested using BsrGI and BstZ17I before transfected into parental Ax2 cells, as shown on the agarose gel. **c** gDNA was prepared from surviving clones after blasticidin selection. PCR screening was carried out using primer sets mentioned above. Gene replacement at the correct sites were detected by primer P1 and P2 with the PCR product at 1368 bp while that of P3 and P4 was at 1091 bp. Parental signal was detected using primer set P2 and P4, with a band at 3121 bp while the size after gene replacement is 3854 bp. Clone 1D5 (clone 1), 3E1 (clone 2) and 3H10 (clone 3) are disruptants and clone 1 is used for main figures.

Fig S6. Verification of *sgf29*$^-$ phenotypes using independent clones.
A. Histone-enriched acid extracts from TSA (4 μM) treated developing Ax2^{bsR} and two further independent clones of *sgf29*$^-$ cells were prepared after 0, 1, 2 or 4 hours of TSA treatment. Samples were resolved by 20% acid-urea electrophoresis and western blots were performed using anti-H3K4me3 and anti-H3 polyclonal antibodies. Position of histone H3 with decreasing net positive charge (0 – 3) are shown on both sides of each blot. **B,C** Cells from three independent *sgf29*$^-$ clones and Ax2^{bsR} controls were washed with KK2 and allowed to develop on solid agar with 1 μM TSA (**B**) or 15 μM SAHA (**C**) for 24 hours as described in Fig. 1a. Images were taken at 24 hours and representative images of three repeats are presented. Clone 1 is used for main figures.

Fig S7. Growth curve of Ax2^{bsR}, *set1*$^-$ and *sgf29*$^-$ cells
Exponentially growing cells were seeded at a density of 7×10^5 cells/ml in HL5 and the cell density was monitored by manual counting over a 3-day period with 12 hours interval. Data was presented as mean ± MSE. n = 3.

Fig S8. Maps of expression vectors used in this study. Please refer to Materials and Methods for detailed cloning steps.

Supplemental Table S1

Primer name	Sequence (5' → 3')	Restriction enzyme sites	Mutation	Annotation
Tudor-F	GCGAATTCAGTAGTGGAAATGGATTCCATGG	EcoRI, NcoI	N/A	Used in cloning of wildtype TTD and the second
Tudor-R	CTCGAGACTAGTCTTATTTAAGGCCAC	XhoI, SpeI	N/A	stage of cloning the F359A/Y366ATTD
Tudor-N-R	AAAGGCGGTGGTATCGGGAGCCAT	N/A	AA to GC	Used in the first stage of PCR to amplify two
Tudor-C-F	CCCGATACCACCGCCTTTGCTCC	N/A	TA to GC	fragment with each carrying one mutation in TTD
DdSgf29_1-854_F	GGATCCAGATCTATGTCCGATGCTC	BamHI, BglII	N/A	
DdSgf29_1-854_R	GTTGCATACCATGGAATCCA	NcoI	N/A	Used to clone 1-854 bp of *Dictyostelium Sgf29*
Sgf29KO_LR_R	GACCAACCTGATCACTCTTTTCTTCATC	N/A	N/A	Used to clone 5' homology arm for the
P1/Sgf29KO_LR_L	CTCACATTCCACCATATACAC	N/A	N/A	*Dictyostelium Sgf29* disruption vector
P2	GAAGTTATCATATGCCGCATGG	N/A	N/A	
P3	ATGCTATACGAAGTTATCCGTGG	N/A	N/A	Primers on BsR cassett
P4/Sgf29KO_RR_R	CATTTCCATATTTATTTGTTGAGTCCC	N/A	N/A	Used to clone 3' homology arm for the
Sgf29KO_RR_F	CATGTTGTGGCCTTAAATAAGTAGTTG	N/A	N/A	*Dictyostelium Sgf29* disruption vector
pSgf29_F	CTCGAGCCAATCCAAACAATTGCTATTACAAGTG	XhoI	N/A	Used to clone the promoter of *Dictyostelium Sgf29*
pSgf29_R	GATAGTAATTGAGCATCGGACAAGATCTTTTAATTG	BglII	N/A	

Fig. S1

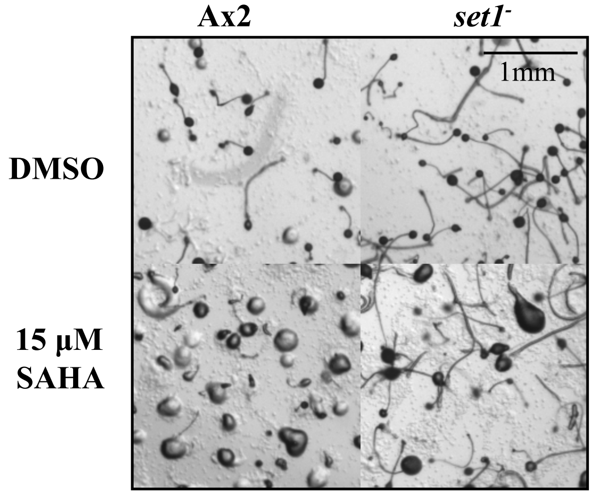

Fig. S2

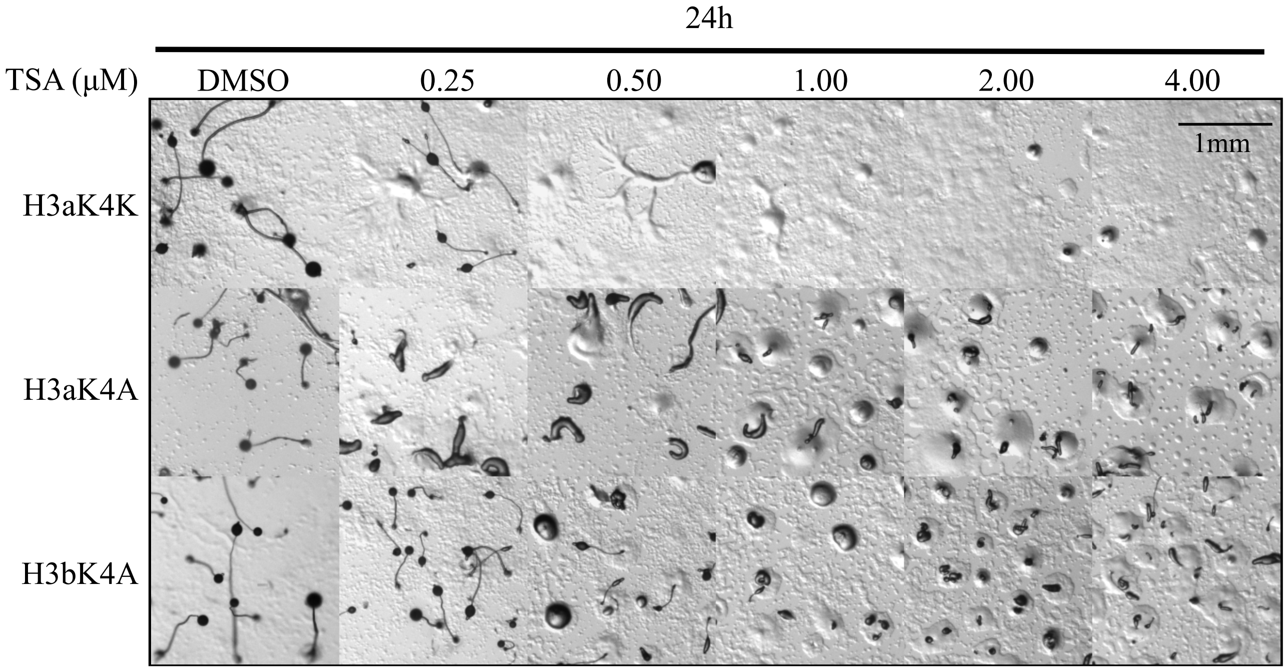

Fig. S3

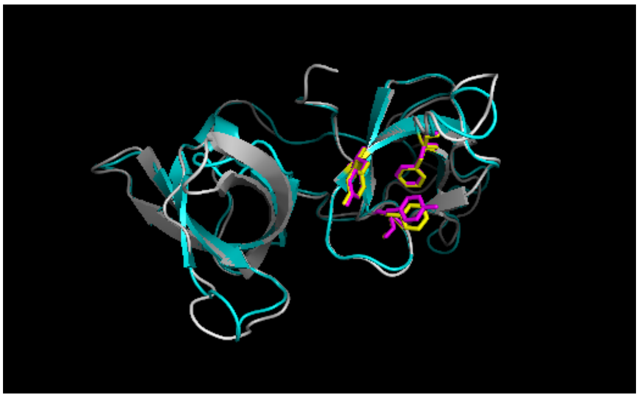

Tandem tudor domain
DdSgf29 V.S. HsSGF29

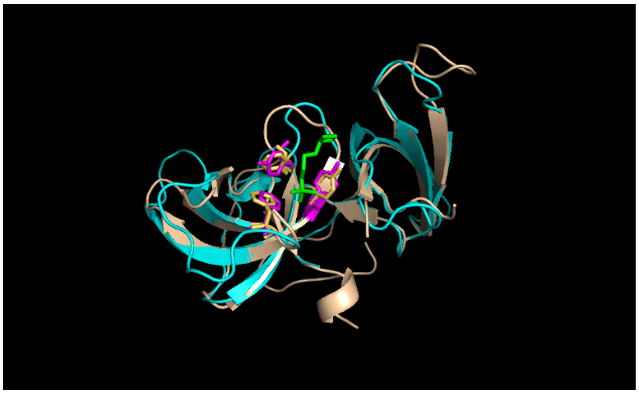

Tandem tudor domain
DdSgf29 V.S. ScSGF29

Fig. S4

A

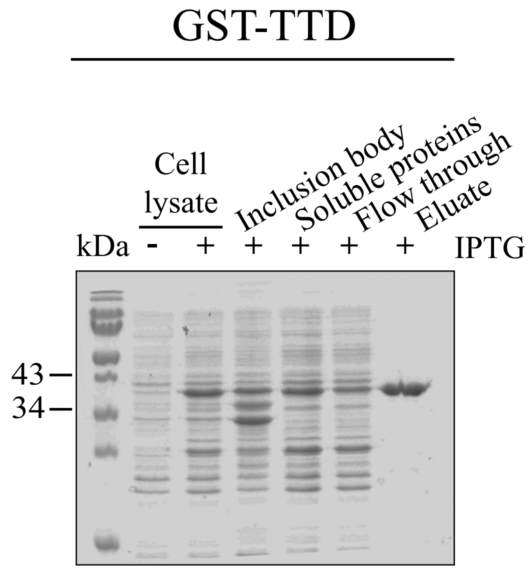
GST-TTD
Cell
lysate
Inclusion body
Soluble proteins
Flow through
Eluate
kDa - + + + + + IPTG
43
34

B

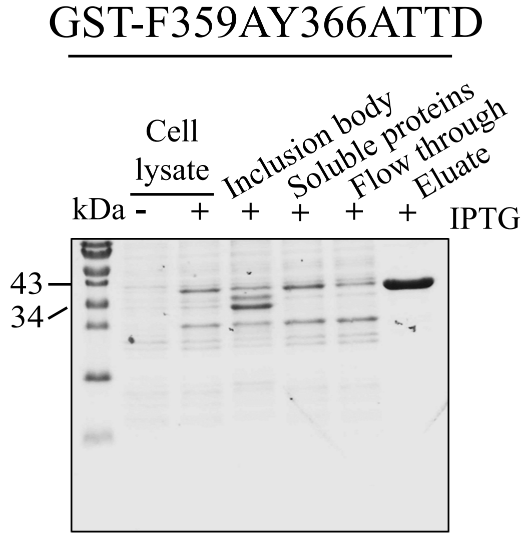
GST-F359AY366ATTD
Cell
lysate
Inclusion body
Soluble proteins
Flow through
Eluate
kDa - + + + + + IPTG
43
34

C

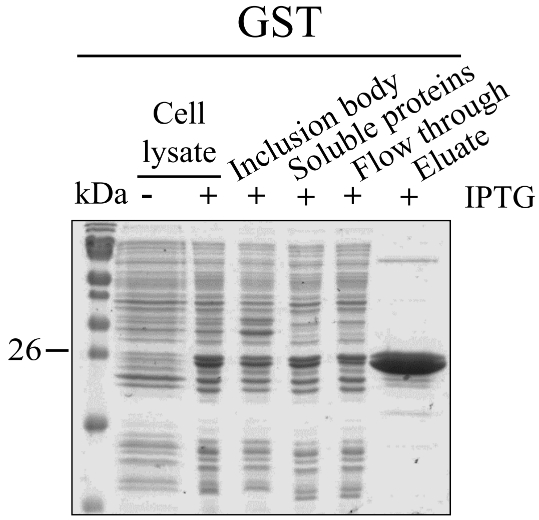
GST
Cell
lysate
Inclusion body
Soluble proteins
Flow through
Eluate
kDa - + + + + + IPTG
26

Fig. S5

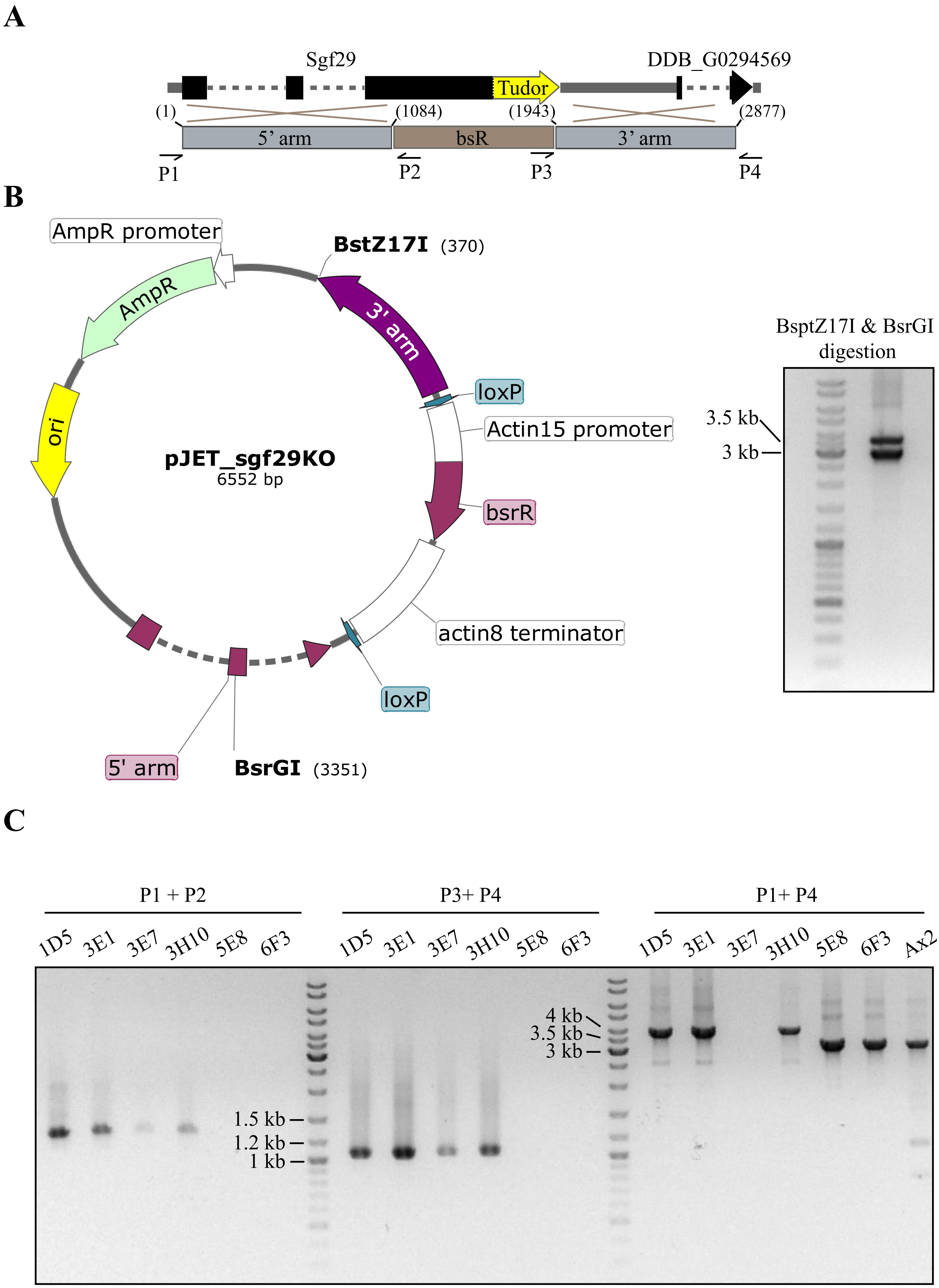
A
Sgf29
DDB_G0294569
Tudor
(1)
(1084)
(1943)
(2877)
5' arm
bsR
3' arm
P1
P2
P3
P4
B
AmpR promoter
AmpR
ori
BstZ17I (370)
3' arm
loxP
Actin15 promoter
pJET_sgf29KO
6552 bp
bsrR
actin8 terminator
loxP
5' arm
BsrGI (3351)
BsptZ17I & BsrGI
digestion
3.5 kb
3 kb
C
P1 + P2
P3+ P4
P1+ P4
1D5
3E1
3E7
3H10
5E8
6F3
Ax2
1.5 kb
1.2 kb
1 kb
4 kb
3.5 kb
3 kb

Fig. S6

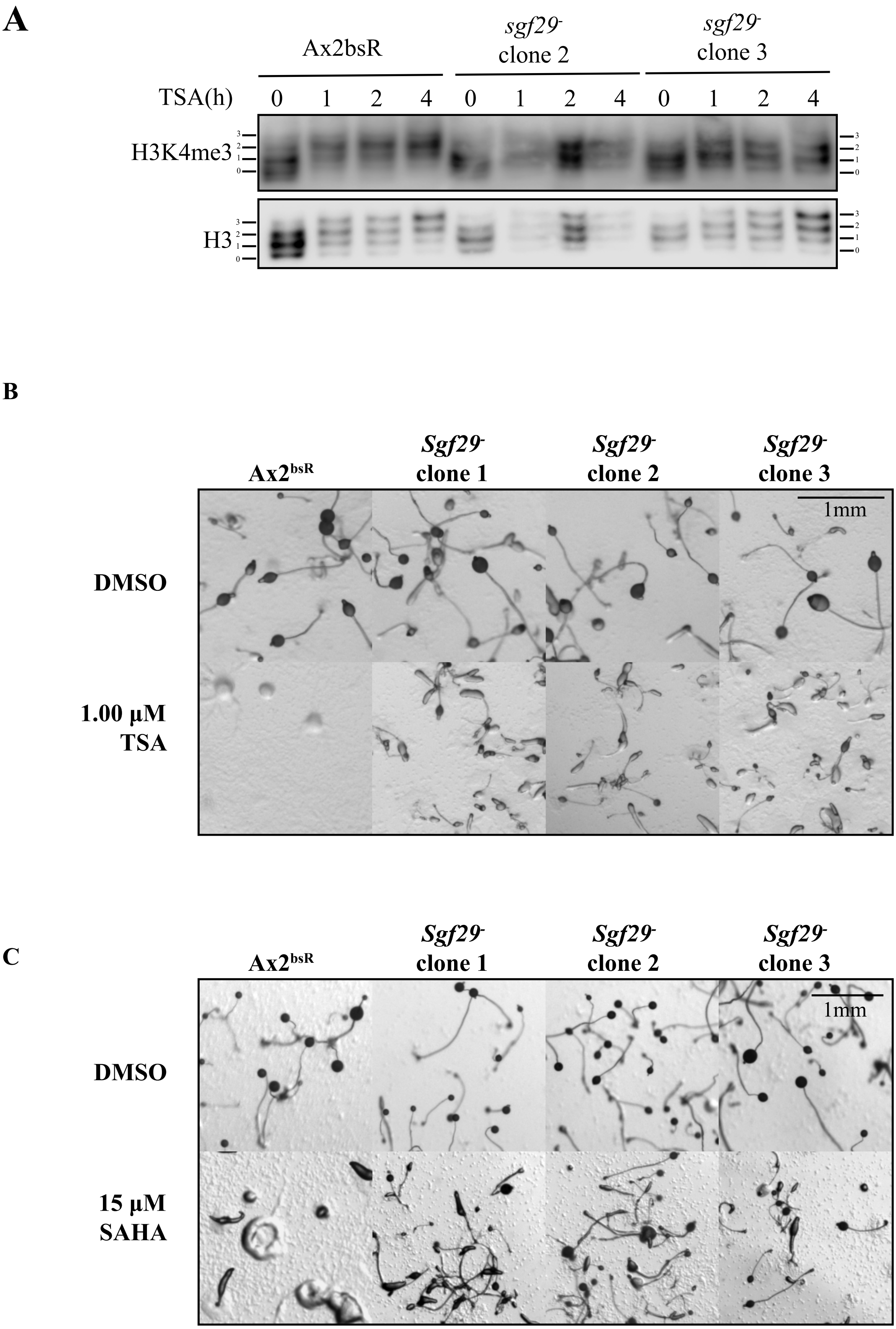
A
Ax2bsR
sgf29- clone 2
sgf29- clone 3
TSA(h)
0 1 2 4 0 1 2 4 0 1 2 4
H3K4me3
H3
3
2
1
0
B
Ax2bsR
Sgf29- clone 1
Sgf29- clone 2
Sgf29- clone 3
1mm
DMSO
1.00 µM TSA
C
Ax2bsR
Sgf29- clone 1
Sgf29- clone 2
Sgf29- clone 3
1mm
DMSO
15 µM SAHA

Fig. S7

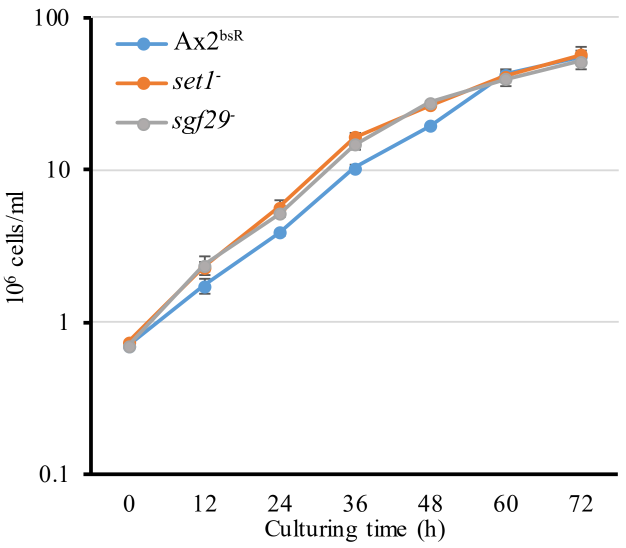

100
10
1
0.1
10^6 cells/ml
Ax2bsR
set1-
sgf29-
0
12
24
36
48
60
72
Culturing time (h)

Fig. S8

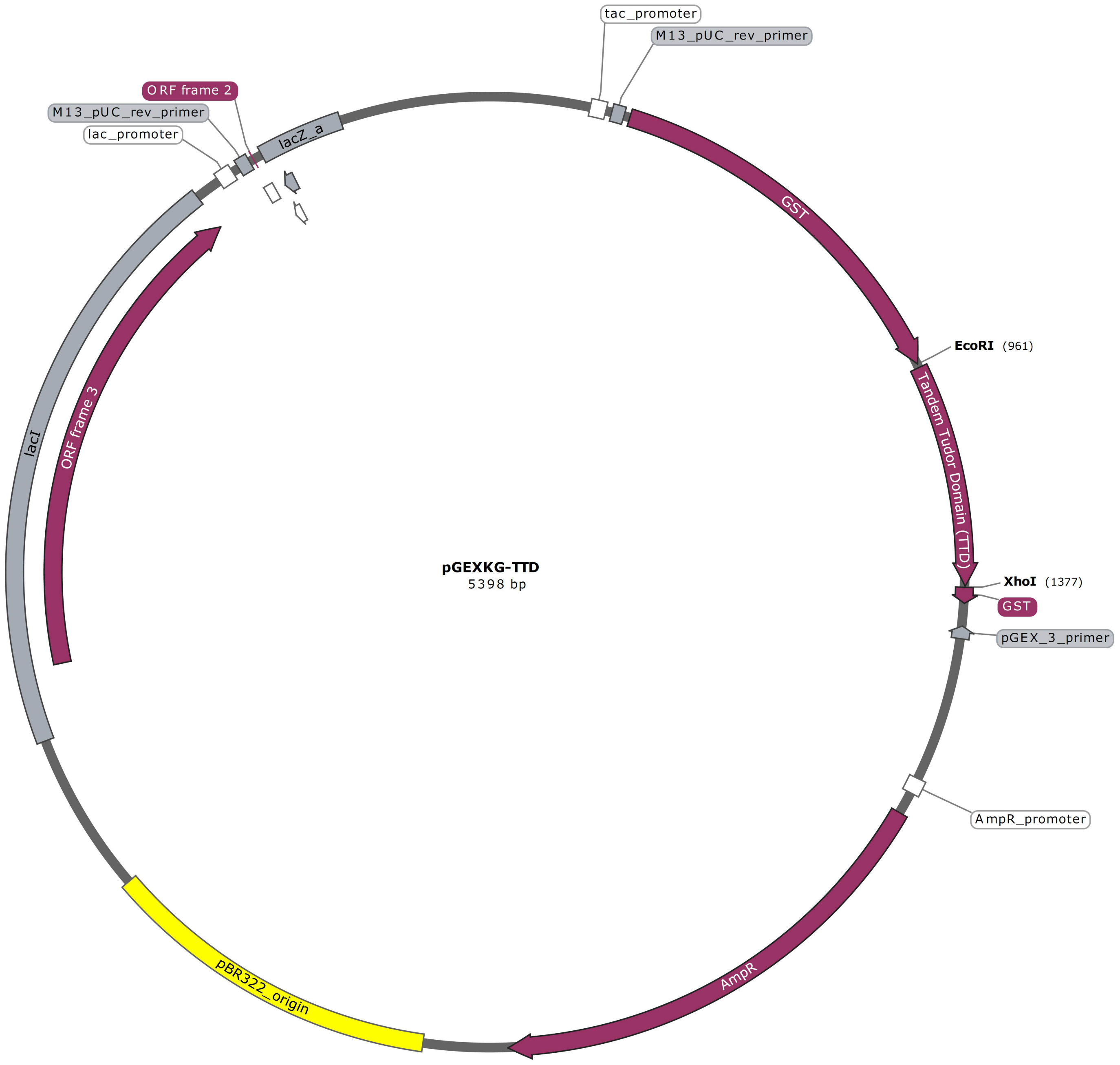
pGEXKG-TTD
5398 bp
tac_promoter
M13_pUC_rev_primer
GST
EcoRI (961)
Tandem Tudor Domain (TTD)
XhoI (1377)
GST
pGEX_3_primer
AmpR_promoter
AmpR
pBR322_origin
lacI
ORF frame 3
lac_promoter
M13_pUC_rev_primer
ORF frame 2
lacZ_a

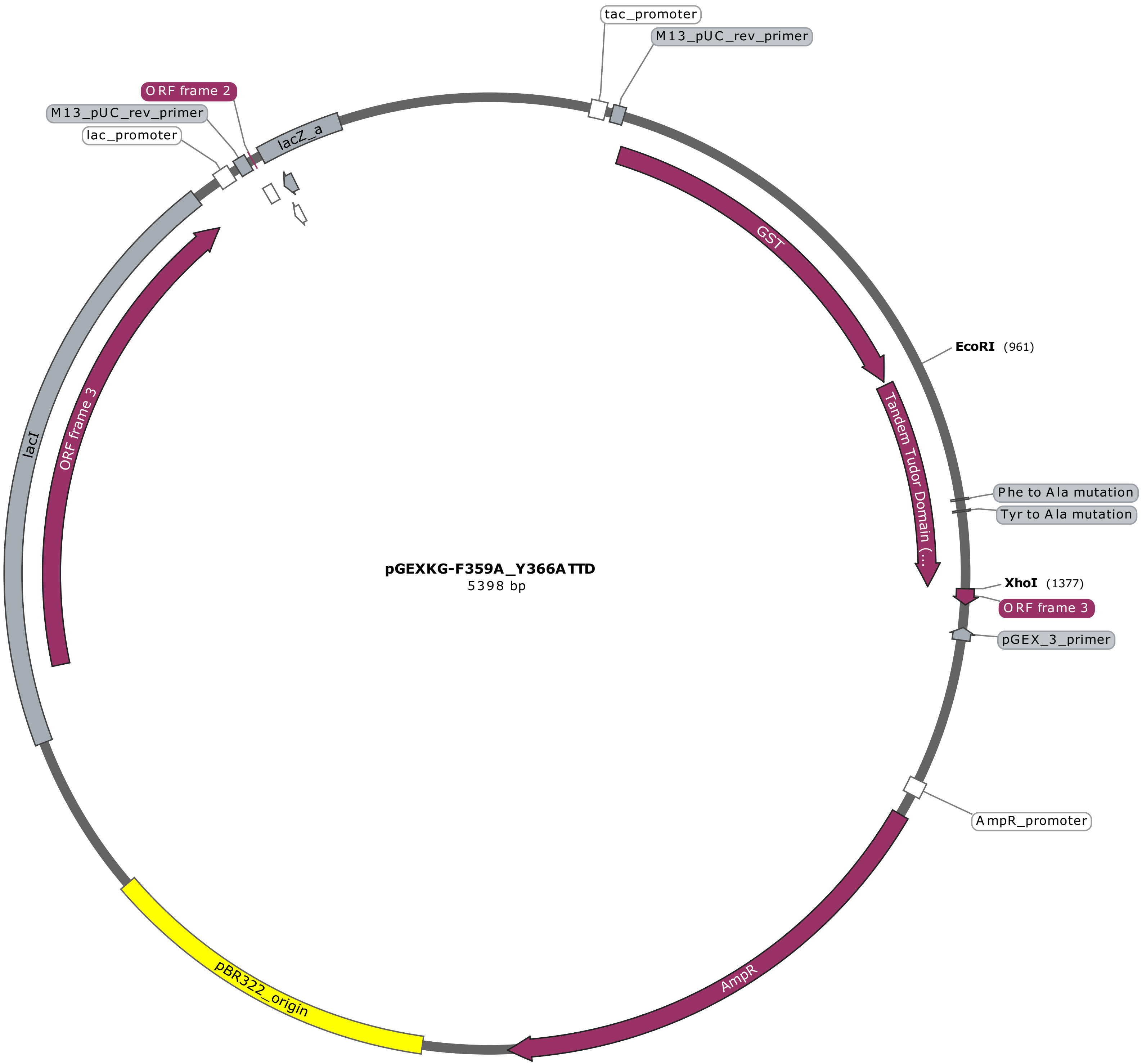

Created with SnapGene®

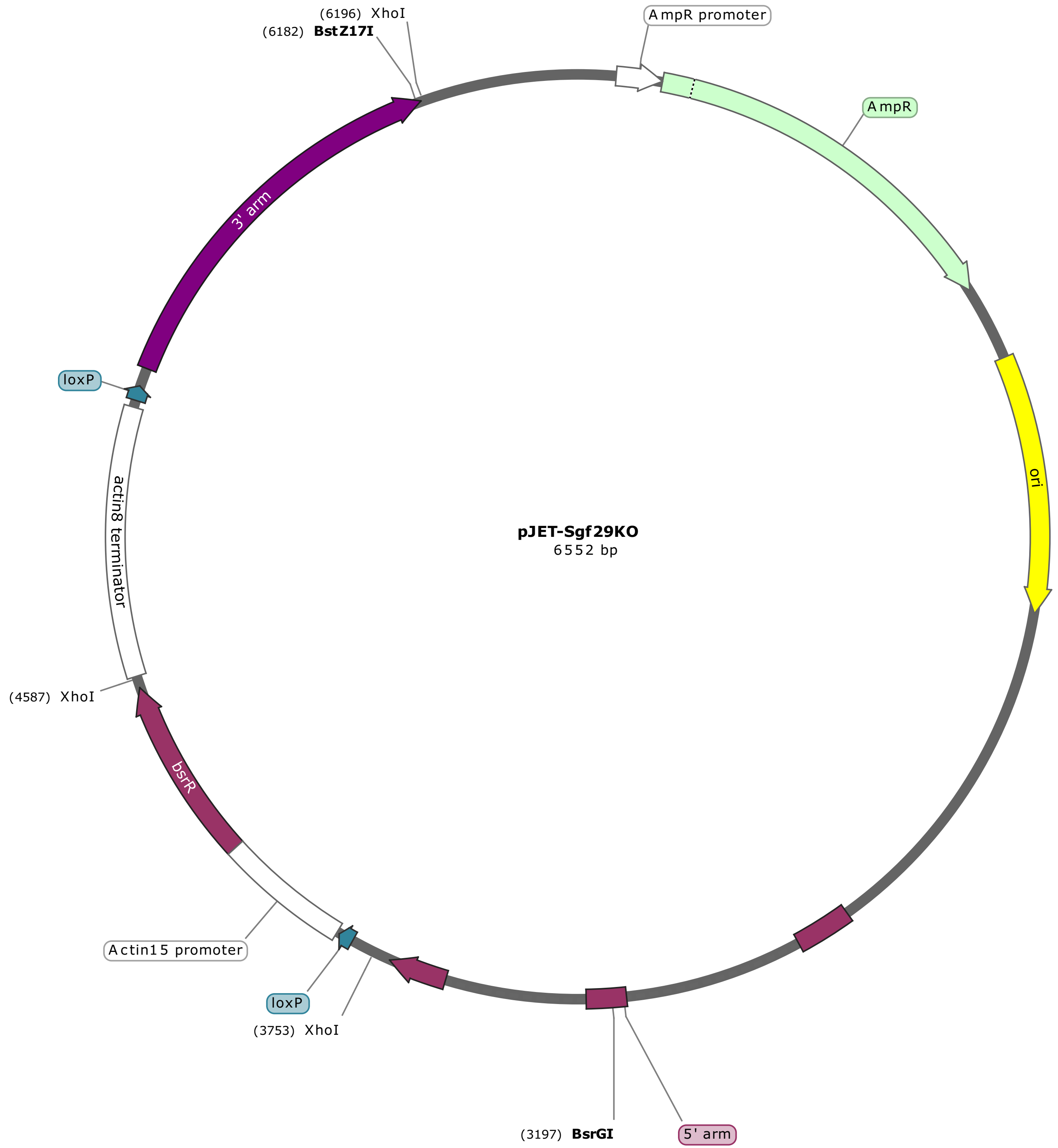

Created with SnapGene®

Cloning of pJET_Sgf29KO.pdf

7033 x 5392

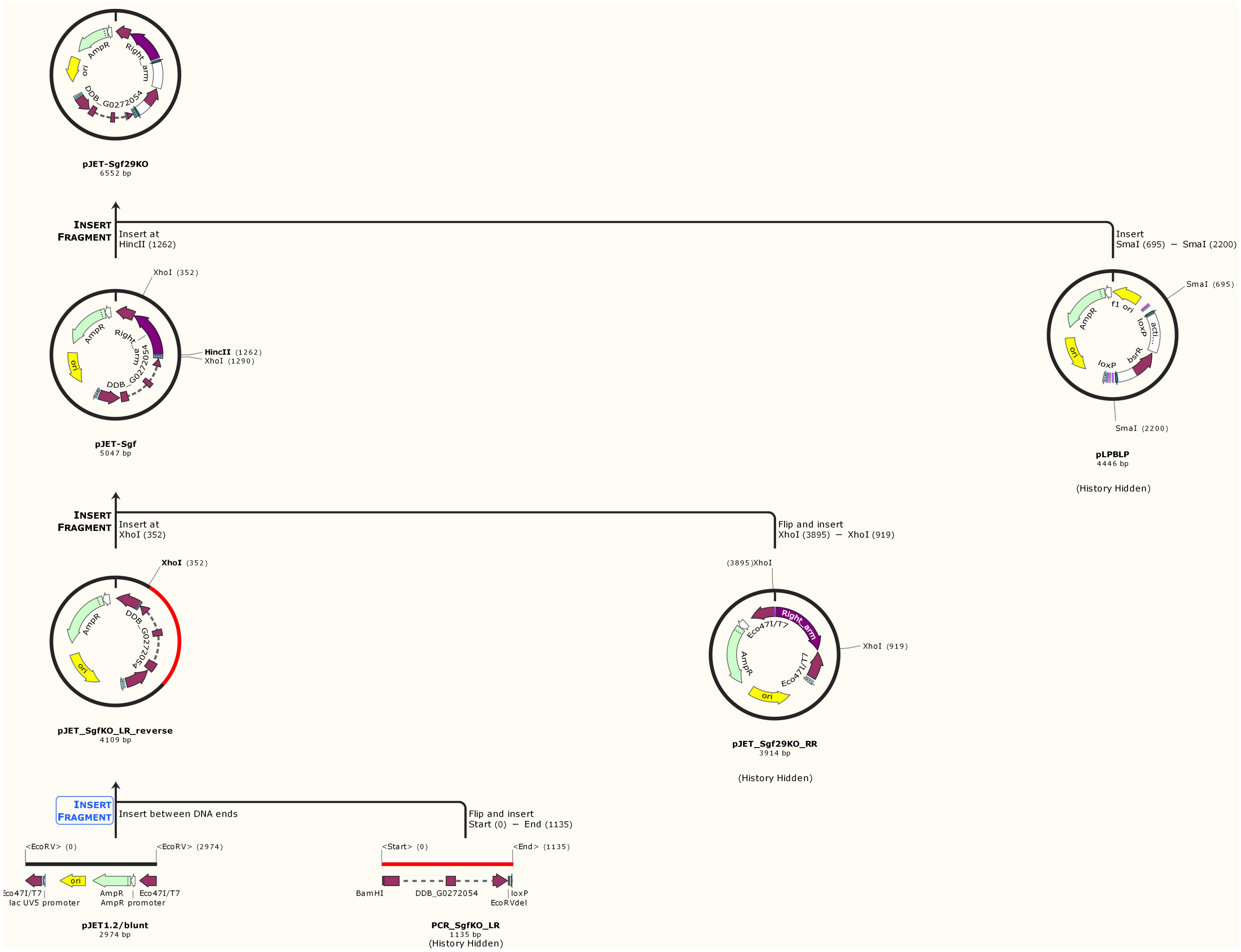

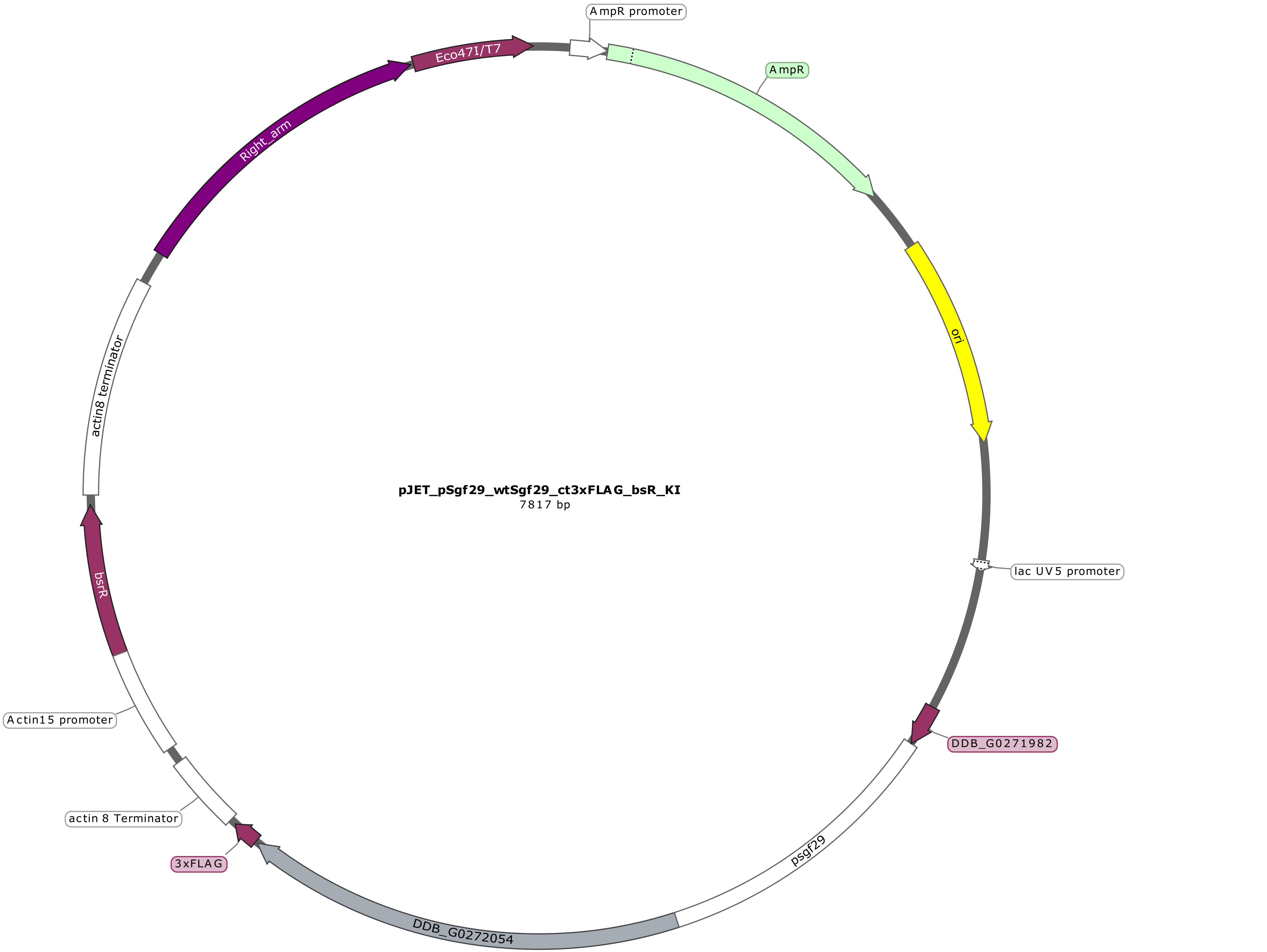

Created with SnapGene®
pJET_pSgf29_wtSgf29_ct3xFLAG_bsR_KI
7817 bp
AmpR promoter
AmpR
ori
lac UV5 promoter
DDB_G0271982
psgf29
DDB_G0272054
3xFLAG
actin 8 Terminator
Actin15 promoter
bsrR
actin8 terminator
Right_arm
Eco47I/T7

Cloning of pJET_pSgf29_Sgf29_ct3xFLAG_bsR_KI.pdf

8640 x 5966

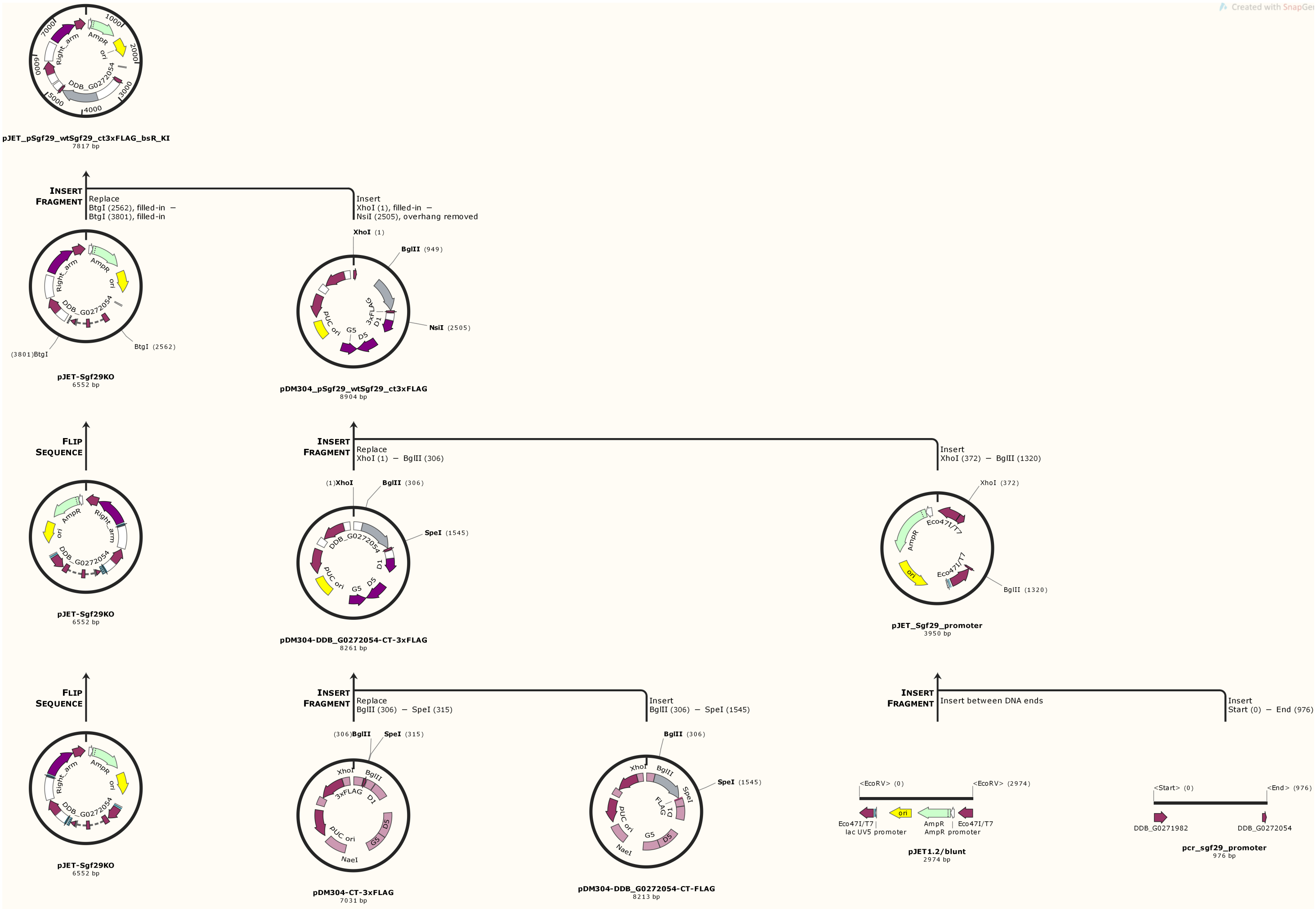

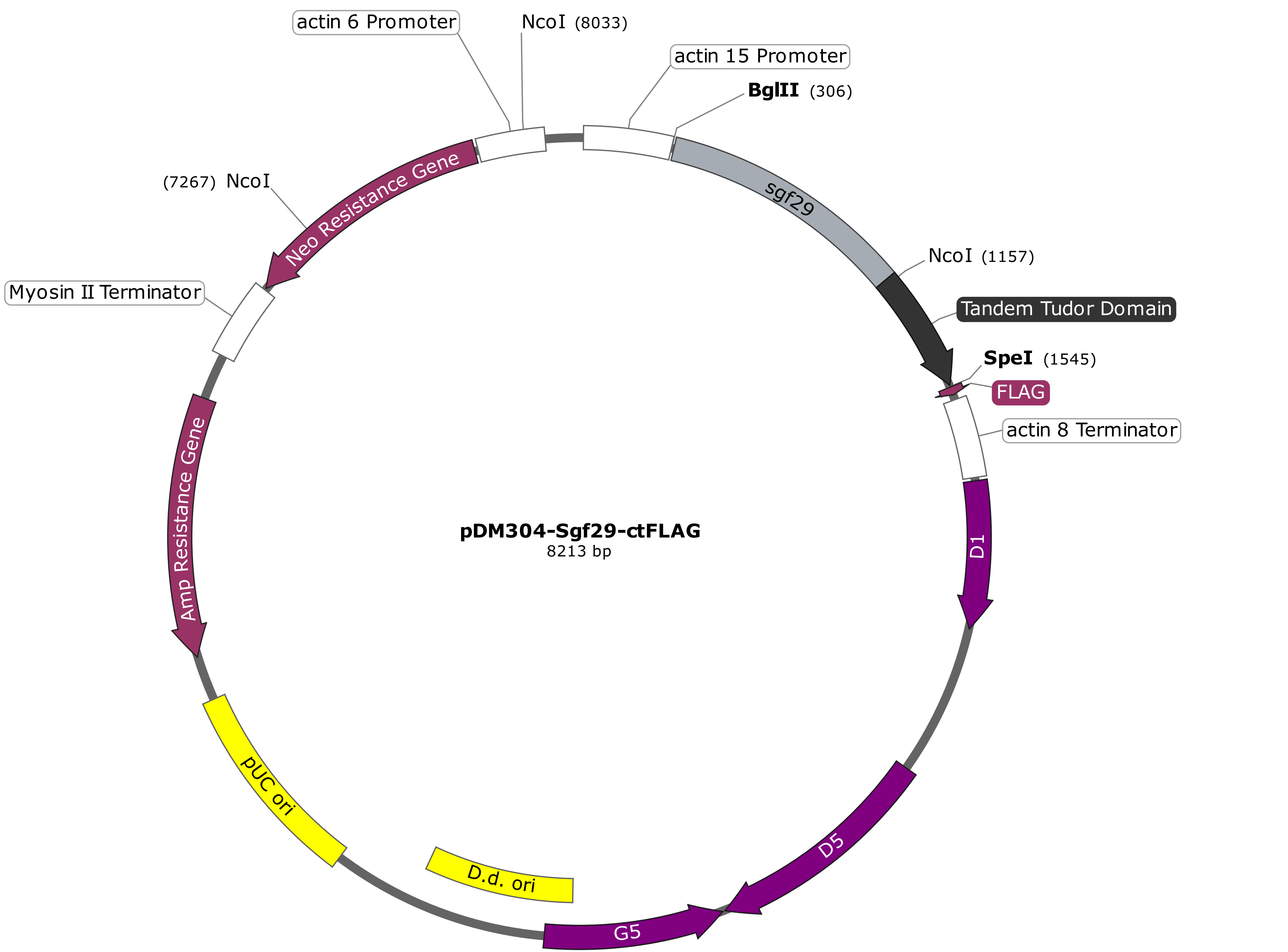
Created with SnapGene®
actin 6 Promoter
NcoI (8033)
actin 15 Promoter
BglII (306)
sgf29
NcoI (1157)
Tandem Tudor Domain
SpeI (1545)
FLAG
actin 8 Terminator
D1
D5
G5
D.d. ori
pUC ori
Amp Resistance Gene
Myosin II Terminator
(7267) NcoI
Neo Resistance Gene
pDM304-Sgf29-ctFLAG
8213 bp

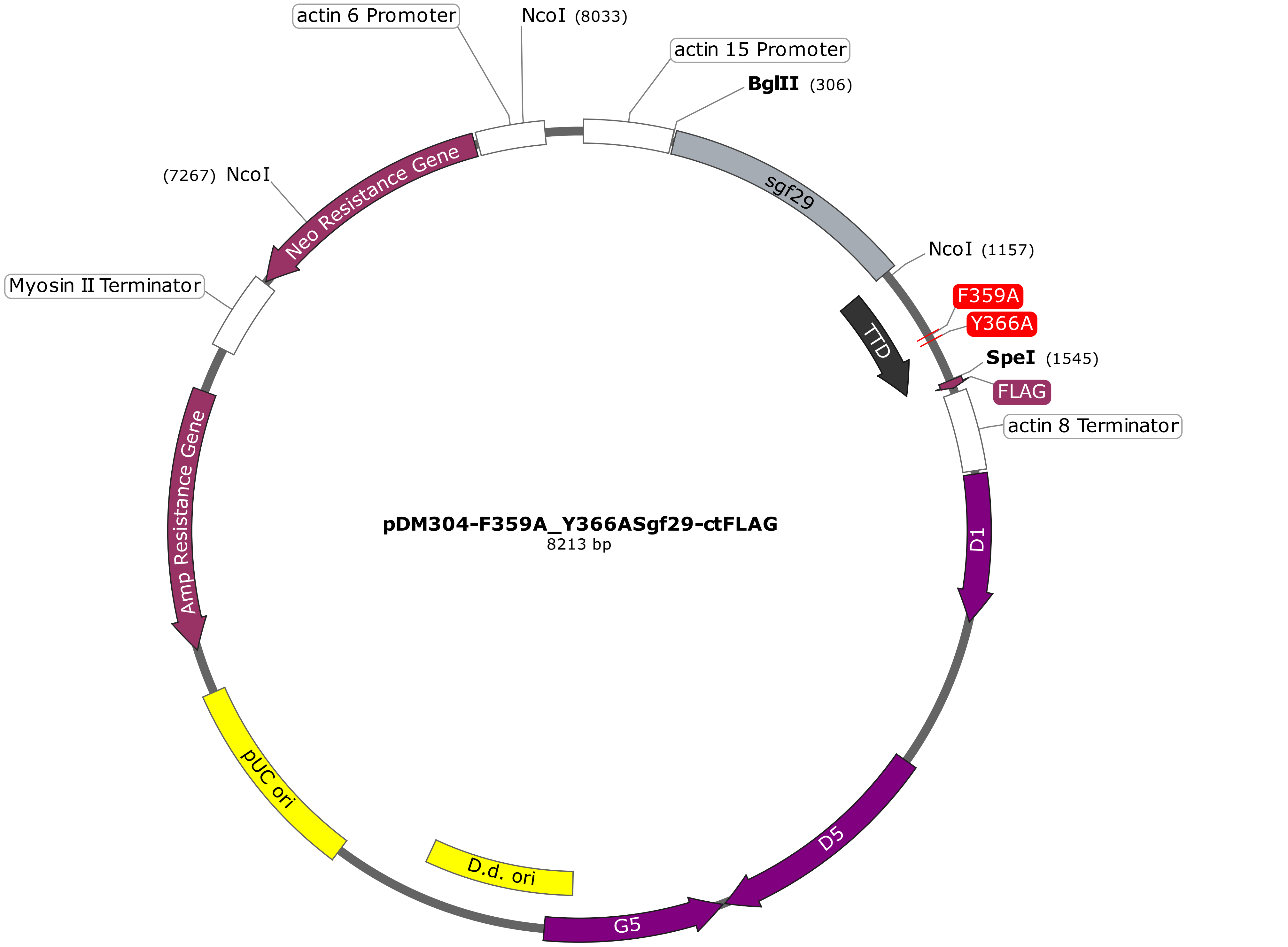
Created with SnapGene®
actin 6 Promoter
NcoI (8033)
actin 15 Promoter
BglII (306)
sgf29
NcoI (1157)
F359A
Y366A
TTD
SpeI (1545)
FLAG
actin 8 Terminator
D1
D5
G5
D.d. ori
pUC ori
Amp Resistance Gene
Myosin II Terminator
(7267) NcoI
Neo Resistance Gene
pDM304-F359A_Y366ASgf29-ctFLAG
8213 bp
